# The βC1 protein encoded in betasatellites promotes begomovirus-whitefly coexistence by subverting vector infestation-induced plant antiviral defenses

**DOI:** 10.1371/journal.ppat.1013907

**Published:** 2026-01-26

**Authors:** Shi-Xing Zhao, Yi-Ming Liu, Su-Dan Wang, Xiao-Wei Wang, Shu-Sheng Liu, Yin-Quan Liu, Li-Long Pan

**Affiliations:** 1 Ministry of Agriculture and Rural Affairs Key Laboratory of Molecular Biology of Crop Pathogens and Insect Pests, Zhejiang Key Laboratory of Biology and Ecological Regulation of Crop Pathogens and Insects, Institute of Insect Sciences, Zhejiang University, Hangzhou, China; 2 The Rural Development Academy, Zhejiang University, Hangzhou, China; The Ohio State University, UNITED STATES OF AMERICA

## Abstract

The transmission of many plant viruses depends on arthropod vectors, which acquire viruses while feeding on infected plants and subsequently inoculate un-infected hosts. Efficient virus acquisition, particularly for persistently transmitted viruses, requires sustained vector feeding on infected plants. However, how vector infestation influences plant-virus interactions and the modulation of these impacts by viral factors remains poorly understood. Here, we show that whitefly infestation on begomovirus-infected plants activates host antiviral defenses through inducing salicylic acid (SA) accumulation. Betasatellites associated with begomoviruses, specifically the βC1 protein encoded therein, suppress these whitefly-induced defenses by interfering with SA accumulation and signaling. Mechanistically, βC1 interacts with *Nicotiana benthamiana* ENHANCED DISEASE SUSCEPTIBILITY 1 (NbEDS1), disrupting its interaction with NbPAD4 to reduce SA accumulation. Additionally, βC1 interferes with the association between NbEDS1 and NbTGA2, thereby attenuating NbTGA2-mediated transcription of SA-responsive genes. Our findings unravel a novel mechanism by which βC1 promotes begomovirus-whitefly compatibility, offering new insights into insect vector-mediated transmission of plant viruses.

## Author summary

The majority of plant viruses are transmitted by insect vectors. Insect vectors acquire viruses when feeding on infected plants and then translocate to un-infected plants to inoculate the viruses. Hence, the feeding of insect vectors on infected plants, or in another word, vector-virus coexistence on plants, determines virus acquisition by insect vectors and in turn vector-mediated virus spread. However, the factors and molecular mechanisms governing vector-virus coexistence on plants are barely known. Using begomoviruses and their whitefly vectors, here we decipher the role of salicylic acid (SA)-signaling pathway and βC1 proteins encoded in begomoviral betasatellites in modulating vector-virus coexistence on plants. Whitefly infestation on virus-infected plants induces SA accumulation and in turn plant defenses against begomoviruses. Begomoviral betasatellites and βC1 proteins encoded therein interfere with whitefly infestation-induced SA accumulation and SA signaling. Mechanistically, βC1 proteins dampen SA accumulation and signaling by subverting plant EDS1-PAD4 interaction and EDS1-TGA2 association, respectively. Our results uncover a novel mechanism by which viral proteins promotes virus transmission by insect vectors.

## Introduction

Plant viral pathogens pose considerable threats to the sustainability of agriculture [1]. The outbreak of these pathogens in the field entails the availability of susceptible plant hosts, environmental conditions conducive to virus infection and efficient inter-plant spread. The majority of plant viral pathogens rely on arthropod vectors such as whiteflies and aphids for inter-plant spread [2,3]. Efficient virus acquisition requires that vectors feed on infected plant hosts for a certain period of time depending on the mode of transmission. While it takes seconds to minutes for the vectors to acquire non-persistently and semi-persistently transmitted viruses, the acquisition of persistently transmitted viruses by vectors usually costs an extensive period of time (from hours to days) [2,3]. During vector infestation on virus-infected plants, active interactions may occur among vector, virus and the host plant [4,5]. Dissecting the tripartite interactions and factors enabling efficient virus uptake by arthropod vectors, will promote our understanding of vector-mediated virus dissemination and, by extension, viral disease epidemics in the field.

Begomoviruses (family *Geminiviridae*) are a group of single-strand DNA viruses that exact a heavy toll on the yield and quality of many crops including tomato, cotton and cassava [6]. Under natural conditions, begomoviruses are exclusively transmitted by whiteflies of the *Bemisia tabaci* complex [7]. The widespread invasion and frequent emergence of large populations of whitefly vectors have fueled the global outbreaks of begomoviruses in the last decades [6,8]. As a group of persistently transmitted viruses, productive acquisition of begomoviruses requires that whiteflies feed on infected plants for at least eight hours [7,9]. Hence, coexistence of whiteflies (often in large populations), begomoviruses and host plants is common in the field, and determines the efficiencies of virus acquisition and subsequent transmission. During coexistence, begomoviruses may modulate plant jasmonates (JA)-mediated defenses, thereby impacting whitefly performance [4,5]. However, whether and how whitefly impact plant-begomovirus interactions during coexistence remain enigmatic.

As a group of piercing-sucking insects, whiteflies feed on plant hosts with their stylets, which probe leaf tissues to extract phloem sap [10]. Saliva containing effectors is secreted into plants and honeydew is dropped onto plant surfaces during whitefly herbivory. Additionally, female whiteflies may produce eggs, which are attached to leaf epidermal cells with pedicels so as to absorb water and nutrients [11]. During whitefly infestation, these factors, alone or in combinations, may significantly modulate plant physiology [12–14]. One of the hallmarkers of whitefly-induced changes in plant physiology is the activation of salicylic acid (SA)-signaling pathway, a key regulator of plant antiviral defenses [13,15–20]. The conserved induction of SA-signaling pathway during whitefly infestation on plants suggests that this antiviral pathway may play a significant role in the coexistence of whitefly and begomovirus on plant hosts. Since the coexistence of whitefly and begomovirus on plant hosts frequently occur, elucidating the factors and mechanisms involved will provide novel insights into whitefly-mediated virus transmission.

In this study, we characterize whitefly infestation-induced plant defenses against begomoviruses and the modulation of these defenses by viral factors during whitefly-begomovirus coexistence on host plants. We first examined the modulation of antiviral defenses and SA-signaling pathway in virus-infected plants by whitefly infestation. Next, we characterized the role of betasatellites and βC1 proteins in subverting whitefly-induced, SA-mediated defenses. Further, we identified plant proteins that were targeted by βC1 proteins and functionally characterized their role in SA-mediated defenses. Finally, we deciphered the mechanism of action of βC1 proteins in dampening whitefly-induced plant antiviral defenses. Our findings dissect a novel mechanism that facilitates the efficient acquisition of begomoviruses by their whitefly vectors.

## Results

### Betasatellites interfere with whitefly infestation-induced, SA-mediated plant antiviral defenses

We first used tomato leaf curl China virus (ToLCCNV), its whitefly vector and the host plant tomato (*Solanum lycopersicum*), to explore the tripartite interactions in virus acquisition. Begomoviruses are frequently associated with betasatellites, which play an important role in suppressing plant immunity [21]. To examine the role of betasatellites, plants were inoculated with either ToLCCNV alone or with tomato leaf curl China betasatellite (ToLCCNB). ToLCCNV infection resulted in stunted growth and ToLCCNV+ToLCCNB induced severe stunted growth, downward leaf curling and puckering in tomato plants (S1 Fig).

**Fig 1:**
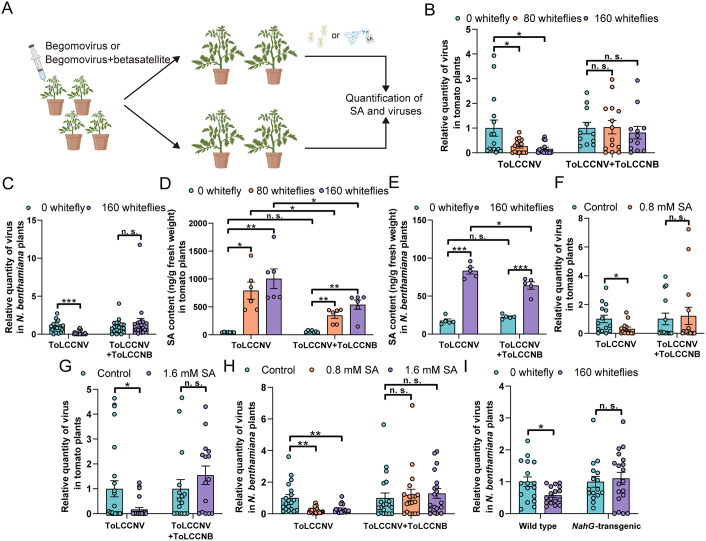
Betasatellites dampen whitefly infestation-induced SA-signaling pathway to sustain begomovirus infection. (A) Schematic representation of experimental design; (B and C) Relative ToLCCNV quantity in ToLCCNV- and ToLCCNV+ToLCCNB-infected tomato (B) or *N. benthamiana* (C) plants that were infested by whiteflies; (D and E) SA content in ToLCCNV- and ToLCCNV+ToLCCNB-infected tomato (D) or *N. benthamiana* (E) plants that were infested by whiteflies; (F-H) Relative ToLCCNV quantity in ToLCCNV- and ToLCCNV+ToLCCNB-infected tomato (F and G) or *N. benthamiana* (H) plants that were treated with SA; (I) Relative ToLCCNV quantity in ToLCCNV-infected wild type and *NahG*-transgenic *N. benthamiana* plants that were infested by whiteflies. N = 11-15 plants for B, 19-20 plants for C, 5-7 samples (2-3 plants per sample) for D and E, 13-25 plants for F and G, 16-20 plants for H-I. Data are mean ± SEM. n. s. stands for no significant difference, *P < 0.05, **P < 0.01, and ***P < 0.001 (two-sided Student’s t test for B-I).

To examine the impact of whitefly infestation on plant-begomovirus interactions, virus-infected plants at 20 days post inoculation were subjected to whitefly infestation or non-infested controls for three days (Fig 1A). The infestation of 80 or 160 whiteflies decreased ToLCCNV quantity in ToLCCNV-infected tomato plants by 73.3% and 84.0%, respectively, yet no significant difference was found between non-infested and whitefly-infested ToLCCNV+ToLCCNB-infected tomato plants (Fig 1B). Similarly, in the model plant *Nicotiana benthamiana*, ToLCCNB abolished whitefly infestation-induced decreases in ToLCCNV quantity (Fig 1C).

As SA plays a key role in plant defenses against viruses and whitefly infestation induces the accumulation of this antiviral hormone [15,20], we profiled free SA and the major SA conjugate (SA-2-*O*-β-D-glucoside, SAG). In ToLCCNV-infected tomato plants, the infestation of 80 and 160 whiteflies significantly increased the contents of SA by a factor of 17.4 and 22.2, respectively (Fig 1D). In ToLCCNV+ToLCCNB-infected tomato plants, 80 and 160 whiteflies increased SA content by a factor of 5.9 and 9.2, respectively (Fig 1D). Whitefly infestation similarly induced SAG accumulation (S2 Fig). Notably, while ToLCCNB did not impact SA and SAG accumulation in non-infested plants, upon whitefly infestations ToLCCNB significantly decreased SA and SAG contents (80 whiteflies per plant: by 56.0% for SA and 44.3% for SAG; 160 whiteflies per plant: by 46.3% for SA and 43.5% for SAG) (Figs 1D and S2). In *N. benthamiana* plants, upon whitefly infestation ToLCCNB decreased SA content by 23.3% (Fig 1E).

Since the response of ToLCCNV and ToLCCNV+ToLCCNB-infected plants to whitefly infestation differed significantly, we next compared whitefly feeding on the two kinds of plants using electrical penetration graph (EPG). When compared to whiteflies feeding on ToLCCNV-infected plants, those on ToLCCNV+ToLCCNB-infected plants displayed similar time to first phloem activity (non-phloem phase before establishing feeding sites) and total duration E1 (watery salivation during the establishment of feeding sites) (S3A-B Figs). However, the total duration of E2 (phloem ingestion) of whiteflies was significantly longer on ToLCCNV+ToLCCNB-infected plants than that on ToLCCNV-infected plants (S3C Fig). These data indicate that whiteflies feed more actively on tomato plants when ToLCCNB is present.

To further determine the role of SA, ToLCCNV and ToLCCNV+ToLCCNB-infected plants were sprayed with SA or ethanol solvents (control) and then ToLCCNV quantity was determined. The treatments of 0.8 or 1.6 mM SA decreased ToLCCNV quantity in ToLCCNV-infected tomato plants by 68.7% and 83.2%, respectively, yet in ToLCCNV+ToLCCNB-infected plants SA treatments did not significantly impact ToLCCNV quantity (Figs 1F-G). Similarly, 0.8 or 1.6 mM SA application significantly decreased ToLCCNV quantity in ToLCCNV-infected *N. benthamiana* plants by 80.0% and 70.1%, respectively, but did not affect ToLCCNV quantity when ToLCCNB was present (Fig 1H).

To assess the role of SA accumulation in whitefly-induced antiviral defenses, *NahG*-transgenic *N. benthamiana* plants wherein SA can not accumulate were used. *NahG* encodes a salicylate hydroxylase that efficiently converts salicylic acid to catechol [22]. Wild type and *NahG*-transgenic *N. benthamiana* plants were inoculated with ToLCCNV and then subjected to whitefly infestation or non-infested control. While whitefly infestation decreased ToLCCNV quantity in virus-infected wild type plants by 42.4%, in *NahG*-transgenic *N. benthamiana* plants no significant difference was found between infested and control plants (Fig 1I).

To examine if the function of ToLCCNB is conserved among betasatellites associated with begomoviruses, we used cotton leaf curl Multan virus (CLCuMuV) and its associated betasatellite (cotton leaf curl Multan betasatellite, CLCuMuB). Whitefly infestation induced SA accumulation in CLCuMuV and CLCuMuV + CLCuMuB-infected plants, yet significantly lower SA accumulated when CLCuMuB was present (S4A Fig). While whitefly infestation and SA treatment significantly decreased CLCuMuV infection in CLCuMuV-infected plants, no significant difference was found in CLCuMuV + CLCuMuB-infected plants (Figs 4B-D).

**Fig 2:**
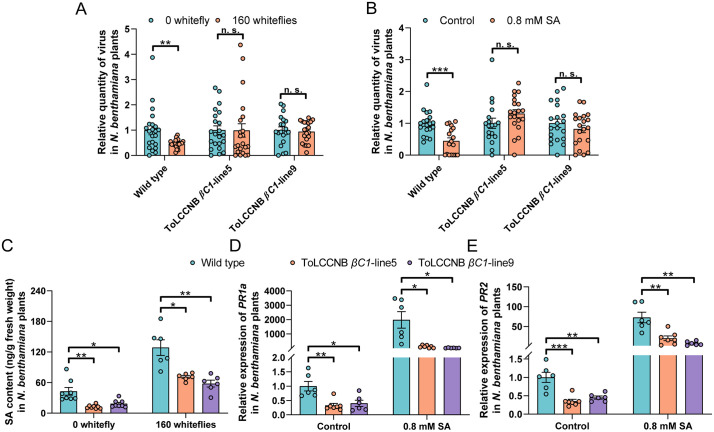
ToLCCNB βC1 abates whitefly infestation and SA-induced antiviral defenses by interfering with SA accumulation and signaling. (A and B) Relative ToLCCNV quantity in ToLCCNV-infected wild type and ToLCCNB *βC1*-transgenic *N. benthamiana* plants that were infested by whiteflies (A) or treated with SA (B); (C) SA content in wild type and ToLCCNB *βC1*-transgenic *N. benthamiana* plants that were infested by whiteflies; (D and E) Relative mRNA level of *PR1a* (D) and *PR2* (E) in wild type and ToLCCNB *βC1*-transgenic *N. benthamiana* plants. N = 18-22 plants for A-B, 6-8 samples (2-3 plants per sample) for C-E. Data are mean ± SEM. n. s. stands for no significant difference, *P < 0.05, **P < 0.01, and ***P < 0.001 (two-sided Student’s t test).

**Fig 3:**
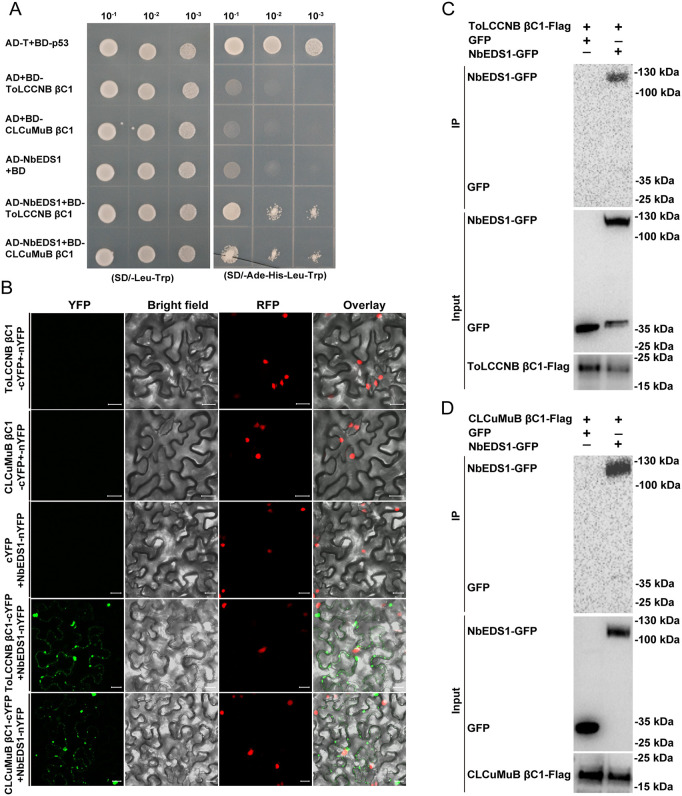
ToLCCNB and CLCuMuB βC1 proteins interact with NbEDS1. (A) Interactions between ToLCCNB βC1 (or CLCuMuB βC1) with NbEDS1 in yeast two-hybrid assay. Yeast cells (strain AH109) were transformed with plasmid combinations (indicated on the left) and then spotted on selective media. (B) Interactions between ToLCCNB βC1 (or CLCuMuB βC1) with NbEDS1 in BiFC assay. ToLCCNB βC1 (or CLCuMuB βC1) was expressed as cYFP-fused protein and NbEDS1 as nYFP-fused protein. cYFP-fused and nYFP-fused proteins were co-expressed in H2B-RFP transgenic *N. benthamiana* plants and fluorescence was examined at two days post inoculation. ToLCCNB βC1 (or CLCuMuB βC1)-cYFP + nYFP and cYFP + NbEDS1-nYFP were used as negative controls. YFP denotes interaction signal and RFP represents nuclei. Bars represent 20 μm. (C and D) Interactions between ToLCCNB βC1 (C) or CLCuMuB βC1 (D) with NbEDS1 in co-IP assay. ToLCCNB βC1 (or CLCuMuB βC1) was expressed as Flag-fused protein and NbEDS1 as GFP-fused protein. Flag-fused and GFP-fused proteins were co-expressed in the leaves of *N. benthamiana* plants and proteins were extracted and subjected to immunoprecipitated with anti-Flag beads. ToLCCNB βC1 (or CLCuMuB βC1)-Flag+GFP was used as negative controls. GFP-tagged proteins were detected with anti-GFP antibodies and Flag-tagged proteins with anti-Flag antibodies.

Taken together, these findings indicate that whitefly infestation on virus-infected plants induces SA accumulation and in turn antiviral defenses, and betasatellites abolish whitefly-induced plant resistance by interfering with SA accumulation and SA-controlled antiviral defenses.

### βC1 proteins mitigate whitefly infestation-induced plant antiviral defenses by interfering with SA accumulation and signaling

Betasatellites primarily encode βC1, a viral pathogenesis determinant and suppressor of plant antiviral defenses [22,23]. We thus sought to determine the role of βC1 in the interference of whitefly infestation-induced plant antiviral defenses by betasatellites. Transgenic *N. benthamiana* plants that ectopically express ToLCCNB *βC1* were constructed and validated with RT-PCR (S5 Fig). Two lines of transgenic plants displayed typical βC1-induced phenotypes including stunted growth and leaf curl (S5A Fig).

While in wild type *N. benthamiana* plants whitefly infestation decreased ToLCCNV quantity by 53.2%, in ToLCCNB *βC1*-transgenic plants whitefly-induced decrease in ToLCCNV quantity was abolished (Fig 2A). Similarly, SA treatment significantly increased plant resistance against ToLCCNV in wild type plants as evidenced by a 55.6% decrease in ToLCCNV quantity, whereas in both ToLCCNB *βC1*-transgenic lines, SA treatment did not induce resistance to ToLCCNV (Fig 2B). To assess the modulation of the SA-signaling pathways by ToLCCNB βC1, SA contents and mRNA level of two SA-responsive genes *PR1a* and *PR2* [24] were examined. ToLCCNB βC1 significantly decreased the contents of SA in non-infested and whitefly-infested plants (Fig 2C). Additionally, in both control and SA treatment, ToLCCNB βC1 significantly decreased the abundance of *PR1a* and *PR2* transcripts (Figs 2D-E).

To examine if CLCuMuB βC1 similarly modulates SA accumulation and signaling, CLCuMuB *βC1*-transgenic *N. benthamiana* plants were constructed and validated (S6A-B Figs). Whitefly infestation and SA treatment induced defenses against CLCuMuV in wild type plants, whereas in CLCuMuB *βC1*-transgenic *N. benthamiana* plants whitefly and SA-induced resistance was abolished (S7A-B Figs). CLCuMuB βC1 significantly decreased SA contents in plants (S7C Fig). In addition, CLCuMuB βC1 significantly downregulated the mRNA level of *PR1a* and *PR2* in control and SA treatment (S7D-E Figs). These data demonstrate that βC1 proteins encoded in betasatellites abolish whitefly-induced antiviral defenses by dampening SA accumulation and downstream signaling.

### βC1 proteins interact with NbEDS1

To probe how βC1 interferes with SA accumulation and signaling, a yeast two-hybrid screen was conducted to identify *N. benthamiana* proteins that interact with ToLCCNB βC1. pGBKT7-ToLCCNV βC1 was used to screen *N. benthamiana* cDNA library ligated to pGADT7. Sequencing of plasmids extracted from positive clones identified several putative ToLCCNB βC1-interacting proteins (S1 Table). ENHANCED DISEASE SUSCEPTIBILITY 1 (Niben101Scf06720g01024.1) was subjected to further analysis due to its well-known role in regulating plant immunity [25]. Expression of NbEDS1-GFP in the leaves of *N. benthamiana* plants revealed that NbEDS1 localized in both nucleus and cytoplasm (S8 Fig).

Yeast two-hybrid was then conducted to examine the interaction between ToLCCNB (or CLCuMuB) βC1 and NbEDS1. Yeast cells transformed with AD-p53 + BD-T (positive control), AD-NbEDS1 + BD-ToLCCNB βC1 and AD-NbEDS1 + BD-CLCuMuB βC1 grew readily on selective media (Fig 3A). No growth of yeast cells transformed with negative controls (AD + BD-ToLCCNB βC1, AD + BD-CLCuMuB βC1 and AD-NbEDS1 + BD) was observed (Fig 3A).

*In vivo* interaction between βC1 proteins and NbEDS1 was examined with bimolecular fluorescence complementation (BiFC) and co-immunoprecipitation (co-IP). ToLCCNB and CLCuMuB βC1 were fused to the C-terminal domain of YFP (cYFP) and NbEDS1 was fused to the N-terminal domain of YFP (nYFP). YFP fluorescence was observed in ToLCCNB βC1-cYFP + NbEDS1-nYFP and CLCuMuB βC1-cYFP + NbEDS1-nYFP treatments, but not in the negative controls (ToLCCNB βC1-cYFP + nYFP, CLCuMuB βC1-cYFP + nYFP and cYFP + NbEDS1-nYFP) (Fig 3B). For co-IP, ToLCCNB (or CLCuMuB) βC1-Flag was co-expressed with NbEDS1-GFP or GFP (negative control) in the leaves of *N. benthamiana* plants. NbEDS1-GFP was specifically detected when co-expressed with ToLCCNB or CLCuMuB βC1-Flag (Figs 3C-D). These results indicate that βC1 proteins encoded in ToLCCNB and CLCuMuB interact with NbEDS1.

### NbEDS1 positively regulates whitefly infestation-induced SA accumulation and signaling and antiviral defenses

To examine the function of NbEDS1, we constructed *NbEDS1*-overexpressing and knockout (*Nbeds1*) *N. benthamiana* plants. *NbEDS1* transgene significantly increased the relative mRNA level of *NbEDS1* in two lines, but did not cause appreciable changes in plant phenotype (S9A-B Figs). Genomic editing using CRISPR/cas9 technique resulted in deletion of one and eleven base pairs, causing frame shift and early termination in *NbEDS1* open reading frame in line4 and line8, respectively (S9C-D Fig).

**Fig 4:**
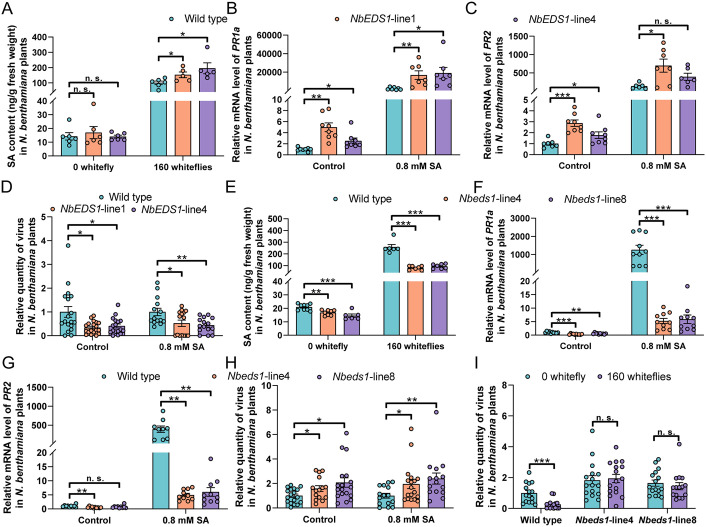
NbEDS1 positively regulates SA accumulation and signaling and is required for whitefly-induced plant antiviral defenses. (A) SA content in wild type and *NbEDS1*-overexpressing *N. benthamiana* plants that were infested by whiteflies; (B-C) Relative mRNA level of *PR1a* (B) and *PR2* (C) in wild type and *NbEDS1*-overexpressing *N. benthamiana* plants that were treated with ethanol (control) or SA; (D) Relative ToLCCNV quantity in ToLCCNV+ToLCCNB-inoculated wild type and *NbEDS1*-overexpressing *N. benthamiana* plants that were treated with ethanol (control) or SA; (E) SA content in wild type and *NbEDS1*-knockout *N. benthamiana* plants; (F and G) Relative mRNA level of *PR1a* (F) and *PR2* (G) in wild type and *NbEDS1*-knockout *N. benthamiana* plants; (H) Relative ToLCCNV quantity in ToLCCNV+ToLCCNB-inoculated wild type and *NbEDS1*-knockout *N. benthamiana* plants; (I) Relative ToLCCNV quantity in ToLCCNV-inoculated wild type and *NbEDS1*-knockout *N. benthamiana* plants that were treated with whiteflies. N = 5-6 samples (2-3 plants per sample) for A, 6-8 samples (2-3 plants per sample) for B and C, 14-19 plants for D, 6-9 samples (2-3 plants per sample) for E, 9-10 samples (2-3 plants per sample) for F and G, 15-18 plants for H and I. Data are mean ± SEM. n. s. stands for no significant difference, *P < 0.05, **P < 0.01, and ***P < 0.001 (two-sided Student’s t test).

While the overexpression of *NbEDS1* did not impact SA content in non-infested plants, upon whitefly infestation SA content was significantly higher in *NbEDS1*-overexpressing plants when compared to wild type control (Fig 4A). The transcripts of two SA-responsive genes *PR1a* and *PR2* were more abundant in *NbEDS1*-transgenic plants in both ethanol (control) and SA treatments except in one case (Figs 4B-C). *NbEDS1* overexpression significantly increased plant antiviral defenses as ToLCCNV and CLCuMuV quantity was significantly lower in *NbEDS1-*overexpressing plants when compared to wild type (Figs 4D and S10A).

Knockout of *NbEDS1* significantly decreased SA content in non-infested and whitefly-infested plants (Fig 4E). The relative mRNA level of *PR1a* and *PR2* was significantly downregulated when *NbEDS1* was knockout in both control and SA-treated plants except in one case (Figs 4F-G). Moreover, knockout of *NbEDS1* impaired plant antiviral defenses as evidenced by increased ToLCCNV and CLCuMuV quantity (Figs 4H and S10B). These results demonstrate that *NbEDS1* positively regulates SA accumulation and signaling and antiviral defenses.

### NbEDS1 is required for whitefly infestation-induced plant antiviral defenses

To examine the role of NbEDS1 in whitefly infestation-induced plant antiviral defenses, wild type and *Nbeds1 N. benthamiana* plants were inoculated with ToLCCNV or CLCuMuV and then subjected to whitefly infestation. In wild type plants, whitefly infestation induced plant antiviral defenses as shown by decreased ToLCCNV and CLCuMuV quantity (Figs 4I and S11). In *Nbeds1* plants, however, whitefly infestation did not induce plant defenses as no significant decrease in ToLCCNV and CLCuMuV quantity was observed (Figs 4I and S11). These data suggest that NbEDS1 is required for whitefly infestation-induced plant defenses against begomoviruses.

### βC1 proteins disrupt NbEDS1 interaction with NbPAD4, a positive regulator of whitefly infestation-induced SA accumulation

EDS1 regulates plant immunity by interacting with phytoalexin deficient 4 (PAD4) or senescence-associated gene 101 (SAG101) [25]. The findings that PAD4 modulates SA biosynthesis in Arabidopsis and *N. benthamiana* plants [26,27] urged us to examine the role of EDS1-PAD4 complex in whitefly infestation-induced SA accumulation.

**Fig 5:**
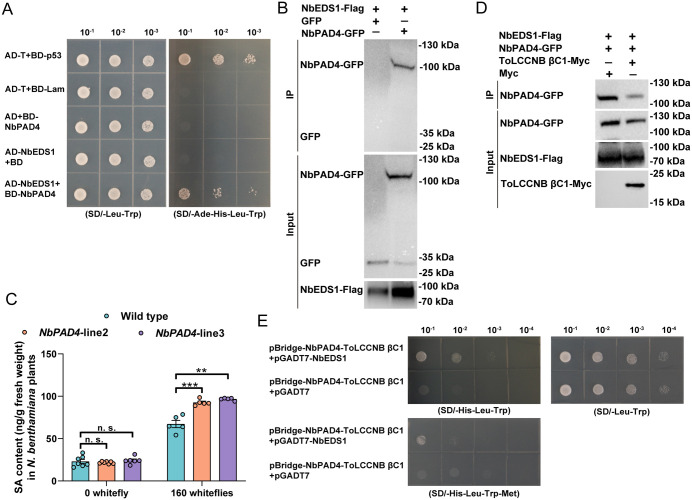
ToLCCNB βC1 disrupts NbEDS1 interaction with NbPAD4, a positive regulator of whitefly infestation-induced SA accumulation. (A and B) Interactions between NbEDS1 and NbPAD4 in yeast two-hybrid (A) and co-IP (B) assay; (C) SA contents in wild type and *NbPAD4*-trangenic *N. benthamiana* plants that were non-infested or infected by whiteflies; (D) Effect of ToLCCNB βC1 on NbEDS1-NbPAD4 interaction in co-IP assay. NbEDS1-Flag+NbPAD4-GFP were co-expressed with Myc empty vector (control) or ToLCCNB βC1-Myc. Two days later, proteins were extracted and subjected to immunoprecipitation with anti-Flag beads. (E) Effect of ToLCCNB βC1 on NbEDS1-NbPAD4 interaction in yeast three-hybrid assay. The coding sequences of *NbPAD4* and ToLCCNB *βC1* were ligated into MCS1 and MCS2 in pBridge vector and recombinant plasmids were co-transformed with pGADT7-NbEDS1 or pGADT7 empty vector (control). Co-transformed yeast cells were spotted on SD/-Leu-Trp medium. Yeast cells were then cultured and transferred to SD/-His-Leu-Trp and SD/-His-Leu-Trp-Met media. N = 5-7 samples (2-3 plants per sample) for C. Data are mean ± SEM. n. s. stands for no significant difference, **P < 0.01, and ***P < 0.001 (two-sided Student’s t test).

We first cloned the coding sequences of *NbPAD4* (Niben101Scf02544g01012.1) and tested its subcellular localization and interaction with NbEDS1. Similar to NbEDS1, NbPAD4 localized in both nucleus and cytoplasm (S8 Fig). In yeast two-hybrid assay, while all yeast transformants can grow on SD/-Leu-Trp medium, only yeast cells containing AD-T + BD-p53 and AD-NbEDS1 + BD-NbPAD4 can readily grow on selective (SD/-Ade-His-Leu-Trp) medium (Fig 5A). In co-IP assay, when co-expressed with NbEDS1-Flag, NbPAD4-GFP was specifically precipitated by NbEDS1-Flag (Fig 5B). These data suggest that NbEDS1 interacts with NbPAD4.

To examine the role of NbPAD4 in whitefly infestation-induced SA accumulation, we generated *NbPAD4*-transgenic *N. benthamiana* plants and validated (S12 Fig). NbPA*D4*-transgene significantly increased the mRNA level of *NbPAD4* (S12A Fig), but did not cause appreciable changes in plant phenotype (S12B Fig). While NbP*AD4* overexpression did not impact SA contents in non-infested *N. benthamiana* plants, upon whitefly infestation *NbPAD4* overexpression significantly increased SA contents (Fig 5C). These findings suggest that NbEDS1 interacts with NbPAD4, a positive regulator of whitefly infestation-induced SA accumulation.

Since βC1 proteins interacted with NbEDS1 and dampen SA accumulation, we hypothesized that βC1 proteins may disrupt NbEDS1-NbPAD4 interaction. Co-IP and yeast three-hybrid assays were thus conducted. NbEDS1-Flag+NbPAD4-GFP were co-expressed with Myc empty vector (control) or ToLCCNB βC1-Myc and then subjected to immunoprecipitation. When compared to control, the expression of ToLCCNB βC1 dramatically decreased the amount of NbPAD4-GFP that were precipitated by anti-Flag beads (Fig 5D).

In yeast three-hybrid, the coding sequences of *NbPAD4* and ToLCCNB *βC1* were ligated into MCS1 and MCS2 in pBridge vector and recombinant plasmids were co-transformed with pGADT7-NbEDS1 or pGADT7 empty vector (control). While genes in MCS1 of pBridge vector can express constitutively, that in MCS2 can only express when methionine was deprived in the medium. Yeast cells containing pBridge-NbPAD4-ToLCCNB βC1 + pGADT7-NbEDS1 grew better on SD/-His-Leu-Trp than that on SD/-His-Leu-Trp-Met, indicating that the expression of ToLCCNB βC1 disrupts NbEDS1-NbPAD4 interaction (Fig 5E). Similarly, CLCuMuB βC1 disrupts NbEDS1-NbPAD4 interaction in co-IP and yeast three-hybrid assay (S13 Fig). These results collectively suggest that βC1 proteins disrupt NbEDS1 interaction with NbPAD4, a positive regulator of whitefly infestation-induced SA accumulation.

### βC1 proteins suppress NbTGA2-mediated SA signaling by dampening NbEDS1-NbTGA2 association

We next explored how βC1 proteins meddle with SA signaling via NbEDS1. We first examined the role of NbPAD4 in regulating the transcription of SA-responsive genes. No significant difference in the relative mRNA level of *PR1a* and *PR2* was found between wild type and *NbPAD4*-transgenic *N. benthamiana* plants in both control and SA treatments (S14 Figs), suggesting that NbPAD4 is not involved in regulating the transcription of SA-responsive genes.

**Fig 6:**
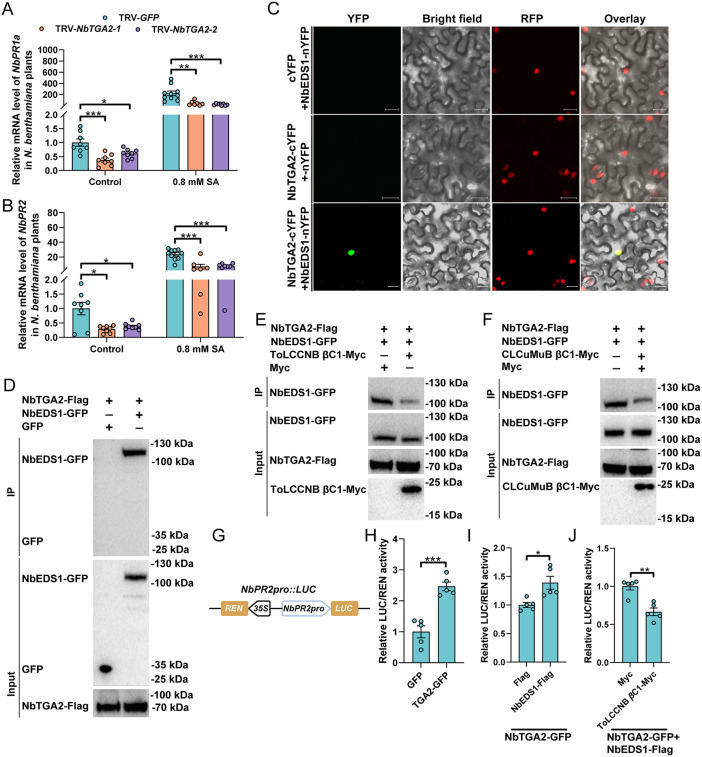
βC1 proteins interfere with NbEDS1-NbTGA2 association and in turn NbTGA2-mediated transcription of SA-responsive genes. (A and B) Relative mRNA level of *PR1a* (A) and *PR2* (B) in TRV-*GFP* and TRV-*NbTGA2*-inoculated *N. benthamiana* plants that were treated with ethanol (control) or SA. (C and D) Association between NbEDS1 and NbTGA2 in BiFC (C) and co-IP (D) assay. In BiFC assay, NbTGA2-cYFP and NbEDS1-nYFP-fused proteins were co-expressed in H2B-RFP transgenic *N. benthamiana* plants and fluorescence was examined at two days post inoculation. YFP denotes interaction signal and RFP represents nuclei. Bars represent 20 μm. In co-IP assay, NbTGA2-Flag was co-expressed with NbEDS1-GFP or GFP in the leaves of *N. benthamiana* plants and proteins were extracted and subjected to immunoprecipitated with anti-Flag beads. GFP-tagged proteins were detected with anti-GFP antibodies and Flag-tagged proteins with anti-Flag antibodies. (E and F) Effect of ToLCCNB βC1 (E) and CLCuMuB βC1 (F) on NbEDS1-NbTGA2 association in co-IP assay. NbTGA2-Flag and NbEDS1-GFP was co-expressed with ToLCCNB βC1-Myc (E) or CLCuMuB βC1-Myc (F) or Myc in the leaves of *N. benthamiana* plants. Proteins were extracted and subjected to immunoprecipitated with anti-Flag beads. GFP-tagged proteins were detected with anti-GFP antibodies, Flag-tagged proteins with anti-Flag antibodies and Myc-tagged proteins with anti-Myc antibodies. (G) Structure of *NbPR2pro::LUC* construct. (H) Effect of NbTGA2 on the activity of *NbPR2* promoter. *NbPR2pro::LUC* were co-expressed with GFP or NbTGA2-GFP in *N. benthamiana* leaves. Two days later, LUC and REN activity was analyzed. (I) Effect of NbEDS1 on NbTGA2-induced activity of *NbPR2* promoter. GFP was used as control. (J) Effect of ToLCCNB βC1 on NbEDS1-NbTGA2-induced activity of *NbPR2* promoter. Myc empty vector was used as control. N = 7–10 samples (2–3 plants per sample) for A-B, 5 samples (5 leaves per sample) for H-J. Data are mean ± SEM. n. s. stands for no significant difference, *P < 0.05, **P < 0.01, and ***P < 0.001 (two-sided Student’s t test).

TGACG-binding TF (TGA) transcription factors such as TGA2 are key regulators of the transcription of SA-responsive genes [28,29]. We cloned *NbTGA2* (Niben101Scf05491g02004.1) based on a recent publication on TGA2 from *N. tabacum*, a close relative of *N. benthamiana* [30]. NbTGA2 localized in the nucleus of *N. benthamiana* cells (S8 Fig). Silencing of *NbTGA2* (S15 Fig) significantly reduced the abundance of *PR1a* and *PR2* transcripts in both control and SA treatments (Figs 6A-B). In GUS staining assay, NbTGA2 significantly increased the expression of GUS that was driven by *PR1a* and *PR2* promoter (2000 bp) (S16 Fig). These findings suggest that NbTGA2 positively regulates the transcription of SA-responsive genes.

We next examine the interplay between NbEDS1 and NbTGA2 and its modulation by βC1 proteins. No direct interaction between NbEDS1 and NbTGA2 was detected as evidenced by the absence of yeast growth in selective media (S17 Fig). However, in BiFC assay, obvious fluorescence in the nucleus was observed in the leaves expressing cYFP-NbTGA2 + nYFP-NbEDS1 (Fig 6C). Similarly, in co-IP assay NbEDS1-GFP was specifically precipitated by NbTGA2-Flag (Fig 6D). We next examined whether βC1 proteins affected NbEDS1-NbTGA2 association. When compared to control (Myc empty vector), the expression of ToLCCNB βC1 and CLCuMuB βC1 dramatically decreased the amount of NbEDS1-GFP that were precipitated by anti-Flag beads (Figs 6E-F). These data suggest that βC1 proteins dampen the indirect interaction between NbEDS1 and NbTGA2.

Finally, we explored whether βC1-NbEDS1-NbTGA2 interplay affected the promoter activity of SA-responsive genes. We sought to clone the promoter of *PR1a* and *PR2* and ligated them into the LUC reporter plasmid. Unfortunately, we only got the reporter plasmid (*NbPR2pro::LUC*) for *NbPR2* even after several attempts and thus used this plasmid for following analysis (Fig 6G). When compared to control (GFP), NbTGA2-GFP significantly increased the activity of *NbPR2* promoter (Fig 6H). The activity of *NbPR2* promoter was significantly higher when NbEDS1 was co-expressed with NbTGA2 (Fig 6I). Importantly, when co-expressed with NbEDS1 and NbTGA2, ToLCCNB βC1 significantly decreased the activity of *NbPR2* promoter (Fig 6J). Protein expression was validated with western blotting (S18 Fig).

Taken together, these results show that NbEDS1 indirectly binds to NbTGA2, thereby promoting NbTGA2-mediated transcription of SA-responsive genes. βC1 proteins interfere with NbEDS1-NbTGA2 association and in turn the transcription of SA-responsive genes.

## Discussion

**Fig 7:**
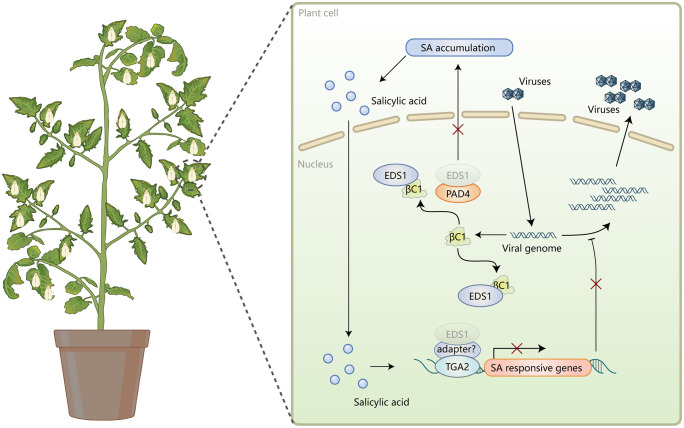
Schematic representation of viral subversion of SA accumulation and signaling during whitefly-begomovirus coexistence on host plants. Upon whitefly feeding on begomovirus-infected plants, EDS1 complexes with PAD4 and thereby mediates SA accumulation. EDS1 indirectly binds to TGA2 transcription factor and in turn activates the transcription of SA responsive genes, thereby dampening virus replication. βC1 proteins encoded in betasatellites associated with begomoviruses interact with EDS1. βC1 proteins interfere with EDS1-PAD4 interaction and in turn SA accumulation. Furthermore, βC1 proteins subvert EDS1-TGA2 association and in turn the transcription of SA responsive genes, thus promoting virus replication.

Productive transmission of persistently transmitted viruses such as begomoviruses requires that insect vectors feed on virus-infected plants for a considerable amount of time so as to efficiently acquire the virus [2,3]. During virus acquisition, insect vectors may interact with the viruses directly or in a plant-mediated manner. Viruses may induce vector antiviral defenses when being ingested and improve vector performance by modulating plant jasmonates-signaling pathway [4,5,31]. However, whether and how insect vectors impact virus infection and the modulation of these impacts by viral factors in the host plants remain unknown. Using begomoviruses and their whitefly vectors, here we unravel a novel aspect of insect vector-virus interactions during coexistence. We show that whitefly infestation on virus-infected plants induces SA accumulation in plants, thereby reducing virus infection in plants. However, betasatellites and βC1 proteins attenuate whitefly infestation-induced SA accumulation and SA-mediated antiviral defenses and in turn sustain virus infection. Mechanistically, βC1 proteins interact with plant EDS1 and interfere with SA accumulation and downstream signaling by dampening EDS1-PAD4 and EDS1-TGA2 interactions, respectively (Fig 7).

Whitefly infestation may significantly modulate many plant biological processes including hormonal signaling pathways [10]. In this study, after we found that whitefly infestation induced antiviral defenses, we focused on SA-signaling pathway as it was conservatively induced by whitefly infestation in many plants. Specifically, activation of SA-signaling pathway by whitefly infestation has been observed in many plants including tomato, tobacco, pepper, soybean, *N. benthamiana* and Arabidopsis [15–20,32]. It should be noted that whitefly infestation may also activate other hormonal signaling pathways such as jasmonates, ethylene and abscisic acid [5,33,34]. Furthermore, these pathways have been shown to modulate plant defenses against viral pathogens [21]. Under this scenario, in addition to SA, these pathways may also play a role in whitefly infestation-induced defenses against begomoviruses. Future investigations may focus on these pathways and thereby advance our understanding of whitefly-begomovirus interactions.

SA is a major regulator of plant-virus interactions that significantly modulate virus intercellular trafficking, long-distance movement and replication in plants [21]. Here we found that whitefly infestation on begomovirus-infected tomato and *N. benthamiana* plants induces SA accumulation and in turn plant antiviral defenses against begomoviruses. The conservativeness of whitefly-induced SA accumulation across plant species suggest that our findings may be recapitulated in other whitefly-begomovirus-plant pathosystems. Moreover, aphids induce SA accumulation and/or the expression of SA downstream genes upon infestation on host plants [35,36]. Under this scenario, aphid infestation on virus-infected plants may also modulate the infection dynamics of the viruses they transmit. Further investigations may explore this issue and resultant findings will unravel new determinants of insect vector-mediated transmission of plant viruses.

Betasatellites associated with begomoviruses and βC1 proteins encoded therein serve as symptom determinants and suppressors of plant defenses including gene silencing and phytohormonal signaling pathways [22,37]. We previously show that upon whitefly-mediated inoculation of begomovirus-betasatellite complexes on un-infected plants, betasatellites and βC1 proteins dampen SA signal transduction and in turn promote begomovirus infection in whitefly-infested un-infected plants [24]. In this study, we uncover a novel function of betasatellites and βC1 proteins in whitefly-begomovirus interaction. We show that during whitefly infestation on virus-infected plants, SA accumulation was reduced when betasatellites and βC1 proteins are present. Notably, whitefly feeds more actively on ToLCCNV+ToLCCNB-infected tomato plants than on ToLCCNV-infected plants. These results suggest that the reduced SA accumulation in begomovirus+betasatellite-infected plants is due to the subversion of whitefly-induced SA accumulation by betasatellites, but not attenuated whitefly feeding on these plants. Additionally, SA-mediated antiviral defense and SA signaling were dampened by betasatellites and βC1 proteins. Since prolonged whitefly-begomovirus coexistence on host plants is a prerequisite for efficient virus acquisition, this function of betasatellites and βC1 proteins sustains viral infection in plants upon whitefly infestation and in turn virus acquisition and transmission by whitefly.

In this study we found that βC1 proteins interact with NbEDS1, thereby dampening SA accumulation and signal transduction. βC1 proteins have been shown to bind to a handful of plant proteins for the subversion of plant biological processes including gene silencing, jasmonate and gibberellin signaling [22,37]. We recently reported that βC1 encoded by tobacco curly shoot betasatellite interacts with heat shock protein 90s (HSP90s) and in turn inhibit plant SA signal transduction [24]. The identification of plant EDS1 as an interactor of βC1 proteins and followed dissection of the manipulation of EDS1 function by βC1 proteins add to our understanding of plant-begomovirus interactions and beyond. Moreover, EDS1 plays a key role in regulating effector-triggered immunity in plants (Lapin et al. 2020). The targeting of EDS1 by βC1 proteins may also significantly modulate plant effector-triggered immunity and should be examined in future investigations.

EDS1 proteins are targeted by several effectors encoded by cellular pathogens, leading to altered interaction between EDS1 and other immune regulators [38,39]. Two bacterial effectors AvrRps4 and HopA1 interfere with EDS1 interactions with the disease resistance proteins RPS4 and RPS6 and the negative immune regulator SRFR1 [38]. A *Phytophthora capsici* effector dampens plant immunity by interacting with EDS1 and dampening EDS1-PAD4 interactions [39]. Here we show for the first time that a group of viral proteins target EDS1 and thereby interfere with its interaction with PAD4 and TGA2, leading to impaired SA accumulation and signaling, respectively.

Members of the EDS1 family including EDS1, PAD4 and senescence-associated gene 101 (SAG101) are central regulators of plant immunity that functions by forming EDS1-PAD4 or EDS1-SAG101 complexes [25]. PAD4 has long been observed as a regulator of pathogen-induced SA accumulation in Arabidopsis and much recently in *N. benthamiana* [26,27]. Here we found that the EDS1-PAD4 complex in *N. benthamiana* positively regulates SA accumulation upon whitefly infestation, uncovering a novel role of this canonical complex in plant interactions with insect herbivores.

Currently, how EDS1-PAD4 module modulates whitefly infestation-induced SA accumulation remain unknown. In plants, SA is synthesized via two routes, namely isochorismate synthase pathway and phenylalanine ammonia lyase pathway [40]. In *N. tabacum*, isochorismate synthase 1 mediates whitefly infestation-induced SA accumulation and the transcription of *isochorismate synthase 1* is regulated by the transcription factor ANAC019 [41]. In *N. benthamiana*, EDS1 and PAD4 interact with the transcription factor WRKY40e, thereby promoting the transcription of *isochorismate synthase 1* [27]. Hence, upon whitefly infestation on plants, EDS1-PAD4 module may interact with transcription factors to regulate the transcription of SA biosynthesis genes and in turn SA accumulation. It is also possible that EDS1-PAD4 module modulates SA degradation, thereby contributing to whitefly infestation-induced SA accumulation. Future investigations may focus on these issues and resultant finding will advance our understanding of plant response to the infestation of phloem-feeding insects.

TGA transcription factors are key regulators of SA-signaling pathway that directly drive the transcription of SA-responsive genes in Arabidopsis [28,29]. The activity of TGA proteins is modulated by a handful of factors including NPR1 [28,42]. Recently, it was found that upon the rise of SA level, AtNPR1 directly recruits AtEDS1 onto the promoter of SA-responsive genes via a physical interaction to stimulate gene transcription [43]. Here we identify NbTGA2, a member of TGA transcription factors in the model plant *N. benthamiana*, and show that its activity as transcription activator is enhanced by NbEDS1. Moreover, we show that βC1 proteins dampen NbEDS1-NbTGA2 association and in turn NbTGA2-medaited transcription of SA-responsive genes. In this regard, βC1 proteins may subvert the direct interactions between NbEDS1 and unknown factors, and in turn NbEDS1-NbTGA2 association. Future investigations may focus on the interplay among βC1 proteins, NbEDS1, NbTGA2 and other factors that leads to impaired transcription of SA-responsive genes.

Taken together, here we show that during coexistence between begomoviruses and their whitefly vectors on host plants, betasatellites and βC1 proteins dampen whitefly infestation-induced SA accumulation and SA signaling, thereby promoting virus infection. Mechanistically, βC1 proteins interact with NbEDS1 and thereby interfere with NbEDS1-NbPAD4 and NbEDS1-NbTGA2 association. These findings unravel a novel aspect of plant virus-insect vector interactions and the mechanisms by which viral proteins promote virus infection during virus-vector coexistence on hosts.

## Materials and methods

### Plants and viruses

Tomato (*Solanum lycopersicum* cv. Moneymaker), *Nicotiana benthamiana* (Laboratory strain), and cotton (*Gossypium hirsutum* cv. ZheMian 1793) plants were used. Tomato and *N. benthamiana* plants were grown in growth chambers at 26 ± 2°C, 60–80% relative humidity with 14/10 h light/dark cycles (light intensity, 200 μmol m^-2^ s^-1^). About one month post sowing, tomato plants at 2–3 true-leaf stage and *N. benthamiana* plants at 5–6 true-leaf stage were used for the inoculation of viral infectious clones. Cotton plants were grown in insect-proof greenhouses to 9–11 true-leaf stage (around two months) under natural lighting at 25 ± 3°C and then used for whitefly rearing.

Genetically modified *N. benthamiana* plants including transgenic and knockout were generated by agrobacterium-mediated transformation by Biorun Co., Ltd. (Wuhan, China). For transgene, the coding sequences of ToLCCNB *βC1* (GenBank accession code: AJ704612), CLCuMuB *βC1* (GenBank accession code: JN968574), *NbEDS1* (Niben101Scf06720g01024.1) and *NbPAD4* (Niben101Scf02544g01012.1) were cloned into the pBWA(V)HS-3xFlag vector and then used for agrobacterium-mediated transformation. Reverse transcription-polymerase chain reaction (RT-PCR) or quantitative PCR was conducted to validate transgenic events. *NbEDS1* knockout *N. benthamiana* plants (*Nbeds1*) were generated with CRISPR/Cas9 technique. Guide RNAs were designed with online tools (https://crispr.cos.uni-heidelberg.de/index.html) and ligated into pHSbdcas9i-S. Genome editing was validated with Sanger sequencing. Transgenic *N. benthamiana* H2B-RFP plants expressing red fluorescent protein fused to the C-terminus of histone 2B were kindly provided by Dr. Xueping Zhou (Institute of Biotechnology, Zhejiang University). *NahG*-transgenic *N. benthamiana* plants were kindly provided by Dr. Xinzhong Cai (Institute of Biotechnology, Zhejiang University).

Two begomoviruses and their associated betasatellites were used. The GenBank accession codes are AJ558119 for tomato leaf curl China virus (ToLCCNV) isolate G18, AJ704612 for tomato leaf curl China betasatellite (ToLCCNB), JN968573 for cotton leaf curl Multan virus (CLCuMuV) isolate GD37, JN968574 for cotton leaf curl Multan betasatellites (CLCuMuB). Infectious clones were provided by Dr. Xueping Zhou (Institute of Biotechnology, Zhejiang University). Agrobacteria containing infectious clones of begomoviruses or betasatellites were first cultured separately until OD600 reached 2.0, and then pelleted and re-suspended in resuspension buffer (10 mM MgCl_2_, 10 mM MES, and 200 μM acetosyringone). Agrobacteria containing infectious clones of begomoviruses were inoculated into plants either alone or in a 1:1 ratio with betasatellites. The OD600 value of agrobacteria containing infectious clones of begomoviruses was kept constant (1.0) between begomovirus and begomovirus+betasatellite inocula. Agroinoculation was conducted with 1 mL syringes. Tomato plants at 20 days post inoculation and *N. benthamiana* plants at 10 days post inoculation were used for whitefly infestation or SA treatment.

### Whitefly rearing and infestation

A culture of MEAM1 whiteflies (mt*COI* GenBank accession code: KM821540) of the *Bemisia tabaci* complex was reared on cotton plants in climate chambers. For whitefly infestation treatment, tomato or *N. benthamiana* plants were placed in insect-proof cages. Whiteflies were then collected and released into cages containing plants. No more than ten plants were placed in one cage, and 80 or 160 whiteflies per plant were released into each cage. Non-infested plants were similarly placed into cages and used as control. Three days later, whiteflies were removed and plants were sampled for the analysis of plant hormone contents and virus quantity.

### Analysis of salicylic acid (SA) and SA-2-*O*-β-D-glucoside (SAG) contents

Analysis of SA and SAG contents was conducted by Nanjing WebiolotechTesting Technology Co., Ltd. (Nanjing, China). The apical three fully-expanded leaves were harvested, and leaves from 2-3 plants were mixed as one sample. Leaves were first grounded into fine power, and then 0.3-0.5 g of power were mixed with 1 mL ethyl acetate containing 8 ng of D4-SA (Sigma, USA). After mixed thoroughly by vortexing, samples were centrifuged at 13,000 rpm for 15 min at 4°C. The supernatants were collected and evaporated using a vacuum concentrator. Dried residues were resuspended in 200 mL of MeOH: H2O (1:1, v/v) and centrifuged at 13,000 rpm for 10 min. The supernatants were then collected, and analyzed with a QSight 420 ultra-performance liquid chromatography-tandem mass spectrometry system (PerkinElmer, USA). SA and SAG contents were calculated with a standard curve made with serial dilutions of SA and SAG standard chemicals (Sigma, USA) containing D4-SA.

### Analysis of whitefly feeding behavior using electrical penetration graph (EPG)

Analysis of whitefly feeding behavior was conducted as per Du et al. (2025) with minor modifications [44]. Briefly, whiteflies (starved for 2 h) were first connected to the Giga-8 DC-EPG amplifier via gold wires (2 cm long and 12.5 μm in diameter) that were glued to whitefly dorsum using a water-soluble silver conductive paint (Colloidal Silver; Wageningen University, the Netherlands). The leaves of ToLCCNV and ToLCCNV+ToLCCNB-infected tomato plants were turned and whiteflies were released on the abaxial surface. The plant electrode (a hard copper wire) was inserted into the soil that was watered prior to analysis. Plants were placed in an electrically grounded Faraday cage in a climate chamber. Whitefly feeding behaviors were monitored continuously for 8 h using an DC-EPG device (EPG Systems, Wageningen University, the Netherlands). Data recording and analysis were conducted with Stylet+ for the Windows software (Wageningen University, the Netherlands).

### Analysis of virus quantity and relative mRNA level

The first apical fully-expanded leaves were collected for the analysis of virus quantity and gene transcripts. DNAs were extracted with the Plant Genomic DNA Kit (Tiangen, China). Total RNAs were extracted using AG RNAex Pro Reagent (Accurate Biology, China) following user manual. cDNA synthesis was conducted with the Evo M-MLV RT Kit with gDNA Clean for qPCR (Accurate Biology, China). qPCR analysis of virus quantity and relative mRNA level was conducted using SYBR Green Premix Pro Taq HS qPCR Kit (Accurate Biology, China) and CFX96 Real-Time PCR Detection System (Bio-Rad, USA). *Actin* was used as the housekeeping gene for the analysis of virus quantity and relative mRNA level. Primers are listed in S2 Table.

### SA treatment

A 1.6 M stock solution of salicylic acid (SA, Sigma, USA) was prepared by dissolving SA in ethanol. SA was diluted with water to 0.8 or 1.6 mM, and ethanol concentration was set at 0.1% in all SA solutions. Ethanol solution (0.1%) was used as control. SA treatment was conducted with a hand sprayer, and approximately 0.5 mL of SA solutions were applied on each plant. Plants were treated once per day for three consecutive days. For the analysis of gene transcripts and virus quantity, plants were sampled at one day post the last spray.

### Yeast two-hybrid and three-hybrid assay

For yeast two-hybrid, the coding sequences of ToLCCNB *βC1*, CLCuMuB *βC1*, *NbPAD4* and *NbTGA2* (Niben101Scf05491g02004.1) were cloned into pGBKT7 vector, and that of *NbEDS1* were cloned into pGADT7 vector. For the screen of *N. benthamiana* proteins that interacted with ToLCCNB βC1, pGBKT7-ToLCCNB βC1 was co-transformed with *N. benthamiana* cDNA library ligated to pGADT7 into *Saccharomyces cerevisiae* AH109 Chemically Competent Cells (Weidi Biology, China) as per the user manual. For the detection of interaction between two proteins, recombinant pGBKT7 plasmids were co-transformed with pGADT7-NbEDS1 plasmids. All yeast transformants were first grown on SD/-Leu-Trp medium, and then transferred to SD/-Ade-His-Leu-Trp medium.

For yeast three-hybrid, the coding sequences of *NbPAD4* and ToLCCNB *βC1* (or CLCuMuB *βC1*) were ligated into the MCS1 and MCS2 of pBridge vector, respectively. The recombinant pBridge plasmids were co-transformed with pGADT7-NbEDS1 into *S. cerevisiae* AH109 Chemically Competent Cells (Coolaber, China) as per the user manual. Yeast transformants were first cultured on SD/-Leu-Trp medium, and then transformed to SD/His-Leu-Trp and SD/His-Leu-Trp-Met media.

### Bimolecular fluorescence complementation (BiFC) and analysis of subcellular localization

The coding sequences of ToLCCNB *βC1*, CLCuMuB *βC1* and *NbTGA2* were cloned and ligated into the p2YC vector to express proteins that were fused with cYFP (C-terminus of YFP, 159–238). The coding sequences of *NbEDS1* were cloned and ligated into the p2YN vector to express NbEDS1 that were fused with nYFP (N-terminus of YFP, 1–158). p2YC and p2YN vectors were kindly provided by Dr. Xueping Zhou (Institute of Biotechnology, Zhejiang University). The recombinant vectors were mobilized into *Agrobacterium tumefaciens* strain EHA105 with electroporation. Equal quantity of agrobacteria containing recombinant p2YC and p2YN plasmids were introduced into the leaves of H2B-RFP transgenic *N. benthamiana* plants. YFP fluorescence was examined at two days post inoculation with a ZEISS LSM 800 confocal microscope (Zeiss, German) (RFP excitation, 561 nm; RFP detection, 580–620 nm; YFP excitation, 514 nm; YFP detection, 520–550 nm).

For the analysis of subcellular localization of NbEDS1, NbPAD4 and NbTGA2, their coding sequences were ligated into pCAMBIA1300-GFP. The recombinant plasmids were introduced into agrobacteria, and the resultant agrobacteria were then used for inoculation into leaves of H2B-RFP transgenic *N. benthamiana* plants. GFP fluorescence was examined at two days post inoculation with a ZEISS LSM 800 confocal microscope (Zeiss, German) (RFP excitation, 561 nm; RFP detection, 580–620 nm; GFP excitation, 488 nm; GFP detection, 505–550 nm).

### Co-immunoprecipitation (co-IP)

The coding sequences of ToLCCNB *βC1*, CLCuMuB *βC1*, and *NbEDS1* were ligated into pBWA(V)HS-3xFlag by Biorun Co., Ltd. (Wuhan, China). *NbTGA2* were ligated into pCAMBIA1300-3xFlag and that of *NbEDS1* and *NbPAD4* were ligated into pCAMBIA1300-GFP. Recombinant plasmids were mobilized into *A. tumefaciens* strain EHA105 using electroporation. Agrobacteria expressing Flag-tagged protein and GFP-tagged protein were cultured and re-suspended separately and mixed in 1:1 ratio. Agrobacteria solutions were introduced into *N. benthamiana* leaves and the leaves were harvested at two days post inoculation. Total proteins were extracted with IP buffer (50mM Tris-HCl, pH 7.5, 50mM NaCl, 10% glycerol, 0.1% Tween 20, 1mM β-mercaptoethanol, 2.5mM imidazole, 1mM DTT, and 1mM PMSF). Flag-tagged proteins and their interactors were isolated with anti-Flag beads (Sigma, USA) and detected with Anti-Flag Tag Mouse Monoclonal Antibody (EarthOx, E022060-01) and Anti-GFP Tag Mouse Monoclonal Antibody (EarthOx, E022030-01).

To examine the impact of ToLCCNB βC1 (or CLCuMuB βC1) on the interaction between NbEDS1 and NbPAD4 or NbTGA2, NbEDS1-Flag+NbPAD4-GFP (or NbTGA2-Flag+NbEDS1-GFP) was co-expressed with either ToLCCNB βC1-Myc or pCAMBIA1300-3xMyc empty vector. Co-IP and followed detection of Flag-tagged proteins and their interactors were conducted as mentioned above. Myc-tagged proteins were detected with Anti-Myc Tag Mouse Monoclonal Antibody (EarthOx, E022050-01).

### Virus-induced gene silencing

Around 300 bp of the coding sequences of *NbTGA2* was ligated into pTRV-RNA2 using primers listed in S2 Table. pTRV-RNA2-GFP was used as control. Recombinant pTRV-RNA2 plasmids were mobilized into *A. tumefaciens* strain EHA105 using electroporation. Agrobacteria containing recombinant pTRV-RNA2 or pTRV-RNA1 were cultured separately, resuspended and mixed to an OD600 of 0.1. Agrobacteria solutions were inoculated into *N. benthamiana* plants. At one week post inoculation, plants were sampled for the analysis of *NbTGA2* transcript level or sprayed with ethanol or SA solution and then subjected to the analysis of mRNA level of SA-responsive genes.

### β-glucuronidase (GUS) histochemical assay and dual luciferase reporter assay

*NbPR1a* and *NbPR2* promoter regions were ligated into the upstream of *GUS* in pBI121 vectors. 35S promoter was similarly introduced into pBI121 vectors and used as positive control. Agrobacteria containing recombinant pBI121 plasmids or pCAMBIA1300-NbEDS1-GFP (or pCAMBIA1300-GFP) were cultured, resuspended and mixed. Agrobacteria solution were inoculated into leaves of *N. benthamiana* plants. Non-inoculated leaves were used as negative control. At two days post inoculation, leaves were harvested and subjected to GUS staining with GUS histochemical kit (Huayueyang Biotechnology, China) as per the user manual. Briefly, leaves were incubated overnight in GUS staining solution (75.5 mM sodium phosphate pH 7.0, 0.1% Triton X-100, 0.05 mM K₃/K₄FeCN, 10 mM Na_2_-EDTA, 20% methanol, and 50 µg/ml X-gluc) at 37°C and then washed with 70% ethanol.

In the dual luciferase reporter assay, *NbPR2* promoter region was cloned into the upstream of the *firefly luciferase* (LUC) gene in the pGreenII0800-LUC vector. Agrobacteria containing pGreenII0800-NbPR2pro-LUC were cultured, resuspended and mixed with agrobacteria expressing GFP, NbTGA2-GFP, Flag+NbTGA2-GFP, NbEDS1-Flag+NbTGA2-GFP, Myc + NbEDS1-Flag+NbTGA2-GFP, and ToLCCNB βC1 + NbEDS1-Flag+NbTGA2-GFP. Agrobacteria solutions were inoculated in the leaves of *N. benthamiana* plants. At two days post inoculation, leaf discs of one centimeter in diameter were collected and subjected to LUC and REN activities analysis with a dual-luciferase assay kit (TransDetect, China) on a FlexStation 3 luminometer (Molecular Devices, USA). Protein expression was validated with western blotting. Relative LUC/REN ratios were calculated as per the manual.

### Statistics

qPCR data of virus quantity and relative mRNA level were calculated as normalized to that of plant *actin* using 2^-ΔCt^ method. All comparisons were conducted using two-sided Student’s independent t-test and differences between treatments were considered significant when *P* < 0.05. To clearly illustrate the differences, the data of virus quantity, relative mRNA level and relative LUC activity in each of the experiments were normalized to that of control. All statistical analyses were conducted using SPSS Statistics 21.0 and EXCEL. All experiments were repeated at least once with similar results.

### Consent for publication

All authors approved the manuscript and gave consent for publication.

## Supporting information

S1 Table List of putative ToLCCNB βC1-interacting proteins. (XLSX)

S2 Table Primers used in this study. (XLSX)

S1 Fig Symptoms of ToLCCNV and ToLCCNV+ToLCCNB infection in tomato plants. (A) Picture of whole tomato plants, and (B-D) enlarged view of apical leaves. Tomato plants were inoculated with agrobacteria containing empty vector (pBINPLUS), infectious clones of ToLCCNV or infectious clones of ToLCCNV+ToLCCNB. Pictures were taken at 20 days post inoculation. ToLCCNV infection resulted in stunted growth and ToLCCNV+ToLCCNB induced severe stunted growth and downward leaf curling and puckering.
(TIF)

S2 Fig ToLCCNB interferes with whitefly infestation-induced SA-2-O-β-D-glucoside (SAG) accumulation. ToLCCNV and ToLCCNV+ToLCCNB-infected tomato plants were subjected to whitefly feeding for three days and then SAG contents were determined. N = 5–6 samples (2–3 plants per sample). Data are mean ± SEM. n. s. stands for no significant difference, **P < 0.01, and ***P < 0.001 (two-sided Student’s t test).
(TIF)

S3 Fig The presence of ToLCCNB promotes whitefly feeding on tomato plants. Whiteflies were allowed to feed on ToLCCNV or ToLCCNV+ToLCCNB-infected tomato plants for an 8-h period. Whitefly feeding behavior was recorded with an electrical penetration graph (EPG) system. The time to first phloem activity (A) indicates the duration of non-phloem phase before establishing feeding sites and total duration E1 period (B) indicates the duration of watery salivation during the establishment of feeding sites. The total duration of E2 period (C) indicates the time of phloem ingestion. N = 8 whiteflies. Data are mean ± SEM. n. s. stands for no significant difference, *P < 0.05 (two-sided Student’s t test).
(TIF)

S4 Fig CLCuMuB dampens whitefly infestation-induced, SA-mediated antiviral defenses. (A and B) SA content (A) and relative CLCuMuV quantity (B) in CLCuMuV- and CLCuMuV + CLCuMuB-infected *N. benthamiana* plants that were infested by whiteflies; (C and D) Relative CLCuMuV quantity in CLCuMuV- and CLCuMuV + CLCuMuB-infected *N. benthamiana* plants that were treated with SA (C: 0.8 mM; D: 1.6 mM) or control. N = 5–6 samples (2–3 plants per sample) for A, 16–24 plants for B-D. Data are mean ± SEM. n. s. stands for no significant difference, *P< 0.05, **P < 0.01, and ***P < 0.001 (two-sided Student’s t test).
(TIF)

S5 Fig Validation of ToLCCNB β C1-transgenic *N. benthamiana* plants. (A) Picture of wild type and ToLCCNB *βC1*-transgenic *N. benthamiana* plants. ToLCCNB βC1-transgene induced stunted growth and upward leaf curl (arrowed). (B) PCR detection of ToLCCNB *βC1* and *NbActin* in plant cDNAs.
(TIF)

S6 Fig Validation of CLCuMuB β*C1*-transgenic *N. benthamiana* plants. (A) Picture of wild type and CLCuMuB *βC1*-transgenic *N. benthamiana* plants. CLCuMuB *βC1*-transgene induced stunted growth, upward leaf curl (red arrowed) and curl of leaf petioles (blue arrowed). (B) PCR detection of CLCuMuB *βC1* and *NbActin* in plant cDNAs.
(TIF)

S7 Fig CLCuMuB βC1 abates whitefly infestation and SA-induced antiviral defenses by interfering with SA accumulation and signaling. (A and B) Relative CLCuMuV quantity in wild type and CLCuMuB βC1-transgenic *N. benthamiana* plants that were first inoculated with CLCuMuV and then treated with whitefly (A) or SA (B); (C) SA content in wild type and CLCuMuB *βC1*-transgenic *N. benthamiana* plants; (D and E) Relative mRNA level of *PR1a* (D) and *PR2* (E) in wild type and CLCuMuB *βC1*-transgenic plants. N = 20–22 plants for A-B, 6–8 samples (2–3 plants per sample) for C, 8–10 samples (2–3 plants per sample) for D-E. Data are mean ± SEM. n. s. stands for no significant difference, *P < 0.05, **P < 0.01, and ***P < 0.001 (two-sided Student’s t test).
(TIF)

S8 Fig Subcellular localization of NbEDS1, NbPAD4 and NbTGA2. GFP, NbEDS1-GFP, NbPAD4-GFP and NbTGA2-GFP were expressed in the leaves of H2B-RFP transgenic *N. benthamiana* plants. Fluorescence was examined at two days post inoculation.
(TIF)

S9 Fig Validation of NbEDS1 transgene and knockout. (A) Relative mRNA level of NbEDS1 in wild type and NbEDS1-transgenic *N. benthamiana* plants; (B) Sequences of *NbEDS1* in wild type and *Nbeds1 N. benthamiana* plants. Red lines indicate deleted base pairs; (C and D) Wild type and *NbEDS1*-overexpressing (C) and knockout (D) plants. N = 4–5 samples (2–3 plants per sample) for A. Data are mean ± SEM. **P < 0.01 (two-sided Student’s t test).
(TIF)

S10 Fig NbEDS1 contributes to plant resistance against CLCuMuV + CLCuMuB. Wild type and *NbEDS1*-transgenic (*NbEDS1*) or *NbEDS1*-knockout (*Nbeds1*) *N. benthamiana* plants were inoculated with CLCuMuV + CLCuMuB. At ten days post inoculation, plants were sampled and subjected to the quantification of CLCuMuV. N = 15–19 plants. Data are mean ± SEM. *P < 0.05, and ***P < 0.001 (two-sided Student’s t test).
(TIF)

S11 Fig NbEDS1 is required for whitefly infestation-induced plant defenses against CLCuMuV. Wild type and *NbEDS1*-knockout (*Nbeds1*) *N. benthamiana* plants were inoculated with CLCuMuV and then subjected to whitefly infestation. N = 15–16 plants. Data are mean ± SEM. **P < 0.01 (two-sided Student’s t test).
(TIF)

S12 Fig Validation of *NbPAD4* transgene. (A) Relative mRNA level of *NbPAD4* in wild type and *NbPAD4*-transgenic *N. benthamiana* plants; (B) Picture of wild type and *NbPAD4*-overexpressing *N. benthamiana* plants. N = 4 samples (2–3 plants per sample) for a. Data are mean ± SEM. ***P < 0.001 (two-sided Student’s t test).
(TIF)

S13 Fig CLCuMuB βC1 interferes with the interaction between NbEDS1 and NbPAD4. (A) Effect of CLCuMuB βC1 on NbEDS1-NbPAD4 interactions in co-IP assay. NbEDS1-Flag+NbPAD4-GFP was co-expressed with CLCuMuB βC1-Myc or Myc empty vector in the leaves of *N. benthamiana* plants. Proteins were extracted and subjected to immunoprecipitation with anti-Flag beads. (B) Effect of CLCuMuB βC1 on NbEDS1-NbPAD4 interactions in yeast three-hybrid assay. Yeast cells were transformed with pBridge-NbPAD4-CLCuMuB βC1 + pGADT7-NbEDS1 or pBridge-NbPAD4-CLCuMuB βC1 + pGADT7 (control) and then grown on SD/-Leu-Trp medium. Yeast cells were then cultured and transferred to SD/-His-Leu-Trp and SD/-His-Leu-Trp-Met media.
(TIF)

S14 Fig NbPAD4 did not impact the transcription of SA-responsive genes. Wild type and *NbPAD4*-overexpressing *N. benthamiana* plants were treated with ethanol (control) or SA and then subjected to the transcriptional profiling of SA-responsive genes. N = 9–10 samples (2–3 plants per sample). Data are mean ± SEM. n. s. stands for no significant difference (two-sided Student’s t test).
(TIF)

S15 Fig Relative mRNA level of NbTGA2 in TRV-GFP and TRV-NbTGA2-inoculated plants. *N. benthamiana* plants were inoculated with TRV1 + TRV-GFP, TRV1 + TRV-NbTGA2–1, TRV1 + TRV-NbTGA2–2. At seven days post inoculation, plants were sampled and subjected to the analysis of *NbTGA2* transcripts. N = 9–10 plants. Data are mean ± SEM. *P < 0.05, and **P < 0.01 (two-sided Student’s t test).
(TIF)

S16 Fig NbTGA2 activates the promoter of SA-responsive genes. The promoter regions (2000 bp) of *NbPR1a* and *NbPR2* were ligated into PBI121 to generate NbPR1apro-GUS and NbPR2pro-GUS. Recombinant plasmids were transformed into agrobacteria and then co-inoculated with GFP or NbTGA2-GFP into the leaves of *N. benthamiana* plants. Leaves inoculated with 35S-GUS were used as positive controls and non-inoculated leaves as negative controls. At two days post inoculation, leaves were harvested and subjected to GUS staining.
(TIF)

S17 Fig NbEDS1 do not directly bind to NbTGA2. Yeast cells (strain AH109) were transformed with plasmid combinations (indicated on the left) and then spotted on selective media.
(TIF)

S18 Fig Validation of protein expression in LUC assay. NbPR2pro-LUC was co-expressed with GFP, NbTGA2-GFP, Flag+NbTGA2-GFP, NbEDS1-Flag+NbTGA2-GFP, Myc + NbEDS1-Flag+NbTGA2-GFP, or ToLCCNB βC1 + NbEDS1-Flag+NbTGA2-GFP in the leaves of *N. benthamiana* plants. At two days post inoculation, leaves were harvested for western blotting.
(TIF)
