# Supplementary figures and images for: The βC1 protein encoded in betasatellites promotes begomovirus-whitefly coexistence by subverting vector infestation-induced plant antiviral defenses
Source: PLoS Pathog. 2026 Jan 26;22(1):e1013907. doi: 10.1371/journal.ppat.1013907 (PMC12858056; doi:10.1371/journal.ppat.1013907)

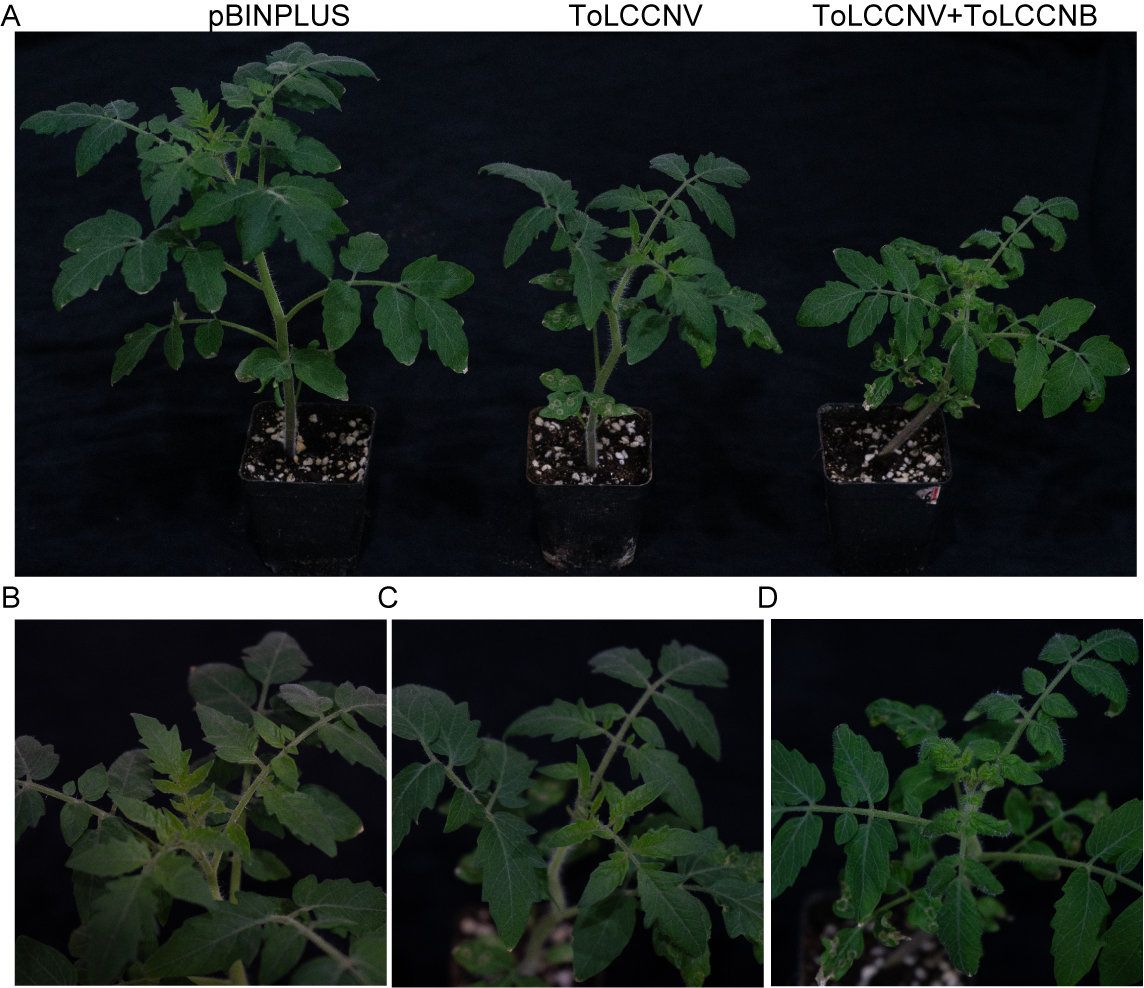

Supplement: S1 Fig — (A) Picture of whole tomato plants, and (B-D) enlarged view of apical leaves. Tomato plants were inoculated with agrobacteria containing empty vector (pBINPLUS), infectious clones of ToLCCNV or infectious clones of ToLCCNV+ToLCCNB. Pictures were taken at 20 days post inoculation. ToLCCNV infection resulted in stunted growth and ToLCCNV+ToLCCNB induced severe stunted growth and downward leaf curling and puckering. (TIF) [file ppat.1013907.s003.tif]

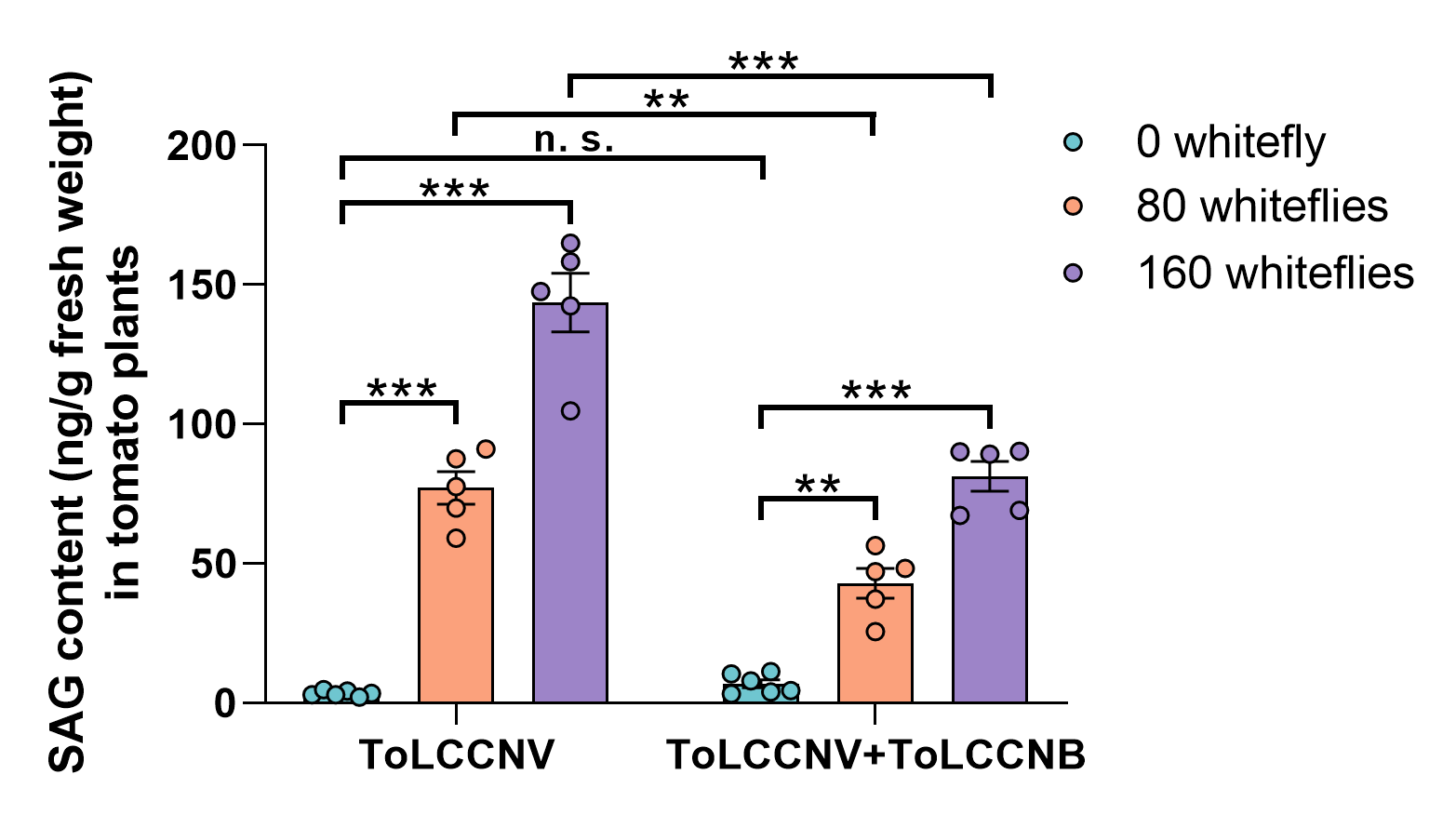

Supplement: S2 Fig — ToLCCNV and ToLCCNV+ToLCCNB-infected tomato plants were subjected to whitefly feeding for three days and then SAG contents were determined. N = 5–6 samples (2–3 plants per sample). Data are mean ± SEM. n. s. stands for no significant difference, **P < 0.01, and ***P < 0.001 (two-sided Student’s t test). (TIF) [file ppat.1013907.s004.tif]

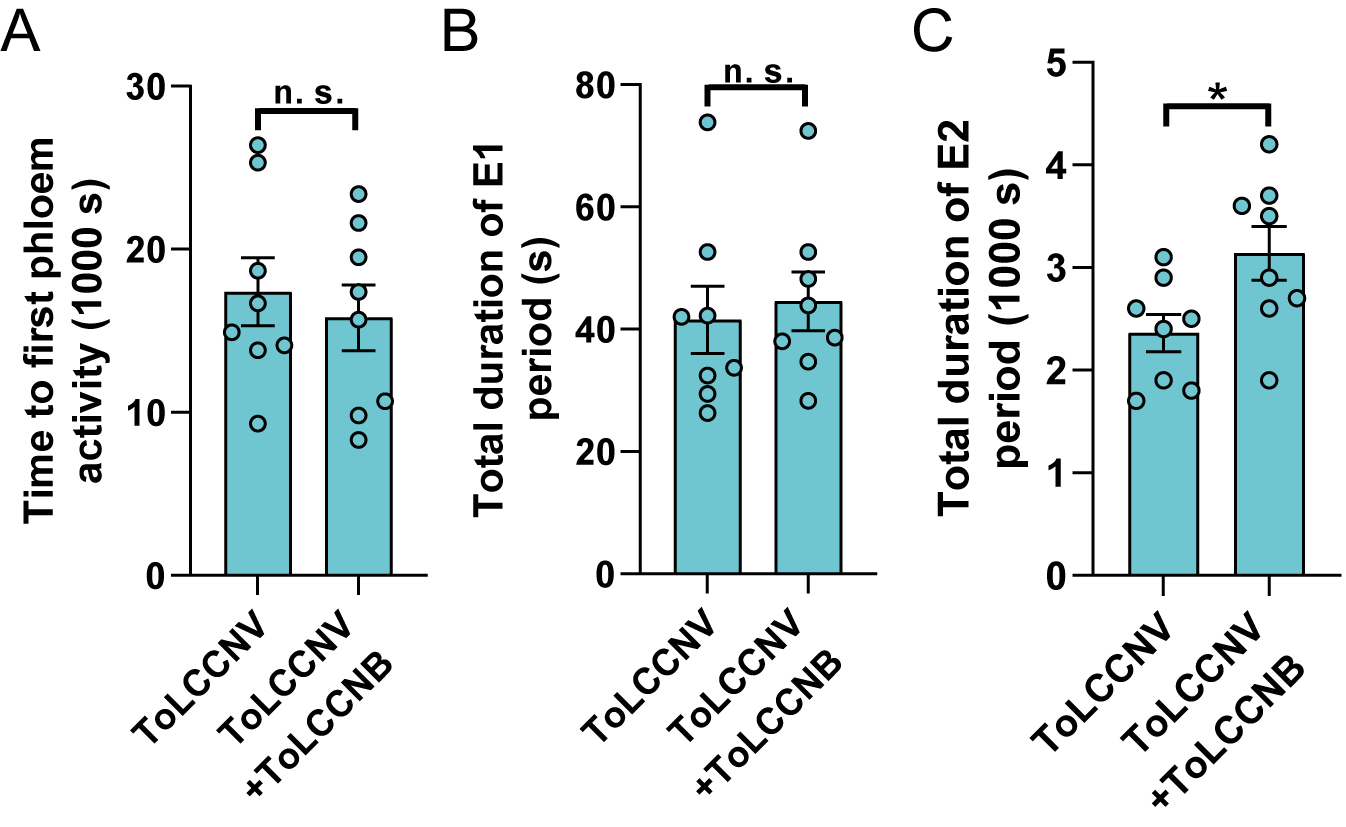

Supplement: S3 Fig — Whiteflies were allowed to feed on ToLCCNV or ToLCCNV+ToLCCNB-infected tomato plants for an 8-h period. Whitefly feeding behavior was recorded with an electrical penetration graph (EPG) system. The time to first phloem activity (A) indicates the duration of non-phloem phase before establishing feeding sites and total duration E1 period (B) indicates the duration of watery salivation during the establishment of feeding sites. The total duration of E2 period (C) indicates the time of phloem ingestion. N = 8 whiteflies. Data are mean ± SEM. n. s. stands for no significant difference, *P < 0.05 (two-sided Student’s t test). (TIF) [file ppat.1013907.s005.tif]

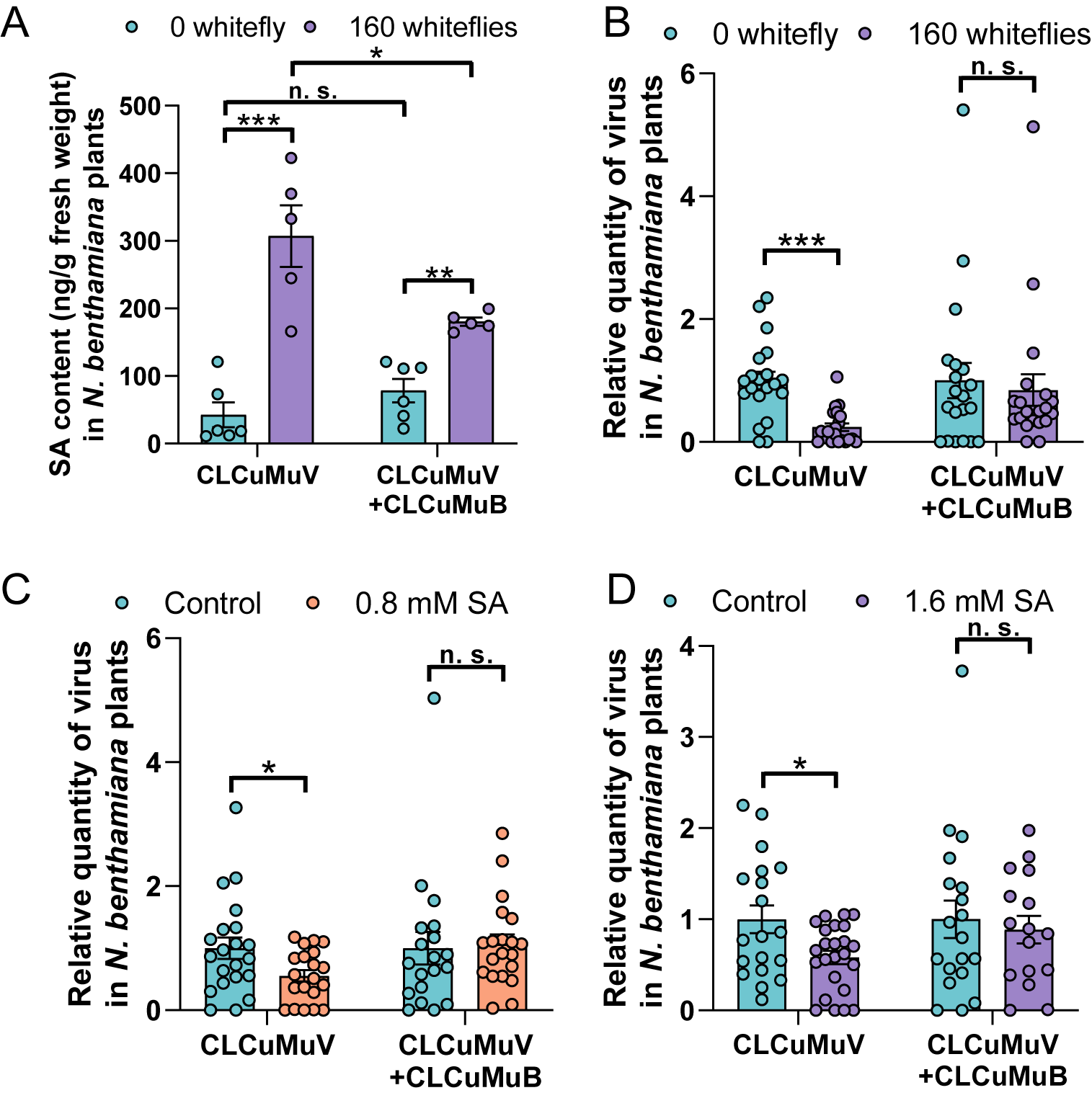

Supplement: S4 Fig — (A and B) SA content (A) and relative CLCuMuV quantity (B) in CLCuMuV- and CLCuMuV + CLCuMuB-infected N. benthamiana plants that were infested by whiteflies; (C and D) Relative CLCuMuV quantity in CLCuMuV- and CLCuMuV + CLCuMuB-infected N. benthamiana plants that were treated with SA (C: 0.8 mM; D: 1.6 mM) or control. N = 5–6 samples (2–3 plants per sample) for A, 16–24 plants for B-D. Data are mean ± SEM. n. s. stands for no significant difference, *P< 0.05, **P < 0.01, and ***P < 0.001 (two-sided Student’s t test). (TIF) [file ppat.1013907.s006.tif]

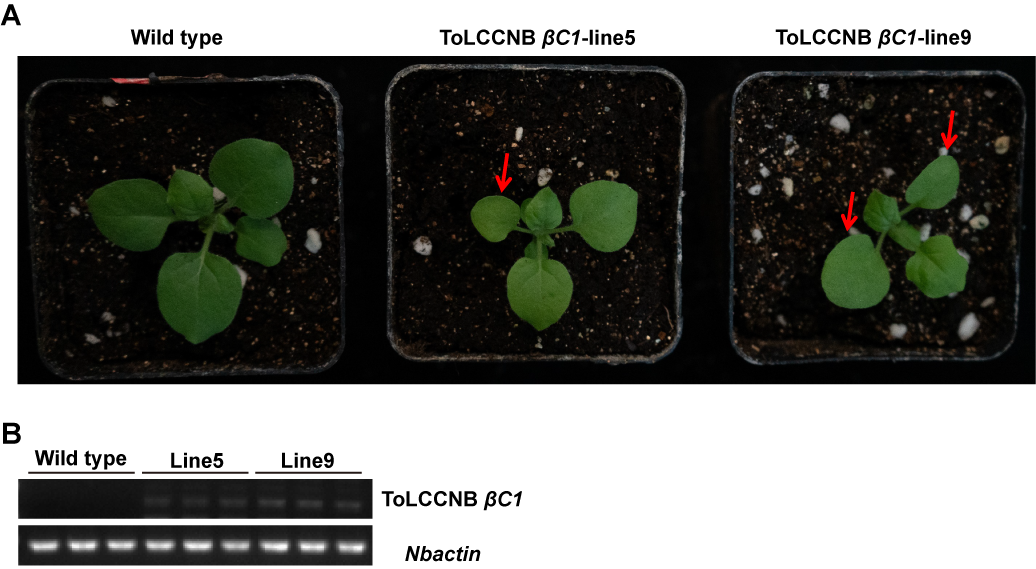

Supplement: S5 Fig — (A) Picture of wild type and ToLCCNB βC1-transgenic N. benthamiana plants. ToLCCNB βC1-transgene induced stunted growth and upward leaf curl (arrowed). (B) PCR detection of ToLCCNB βC1 and NbActin in plant cDNAs. (TIF) [file ppat.1013907.s007.tif]

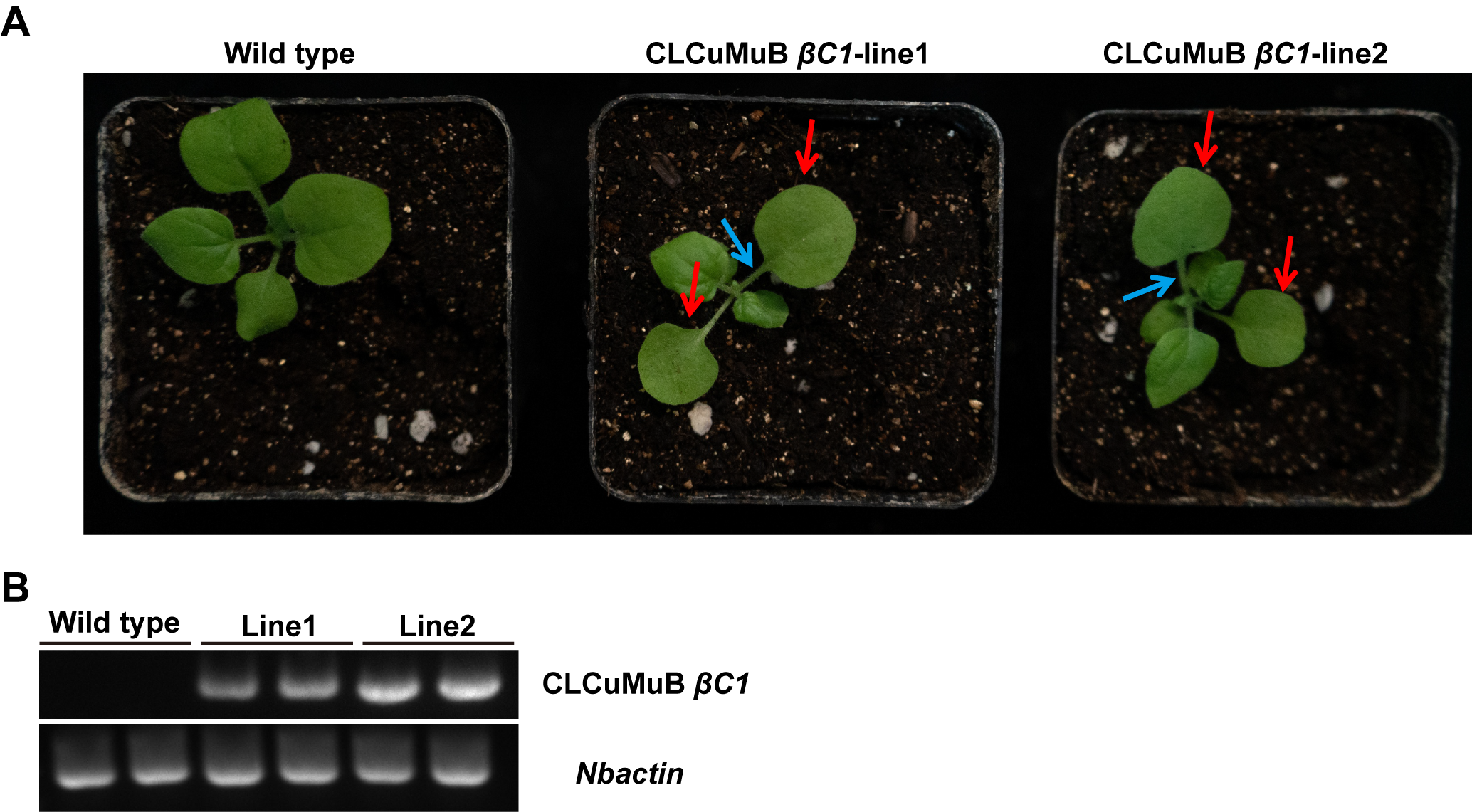

Supplement: S6 Fig — (A) Picture of wild type and CLCuMuB βC1-transgenic N. benthamiana plants. CLCuMuB βC1-transgene induced stunted growth, upward leaf curl (red arrowed) and curl of leaf petioles (blue arrowed). (B) PCR detection of CLCuMuB βC1 and NbActin in plant cDNAs. (TIF) [file ppat.1013907.s008.tif]

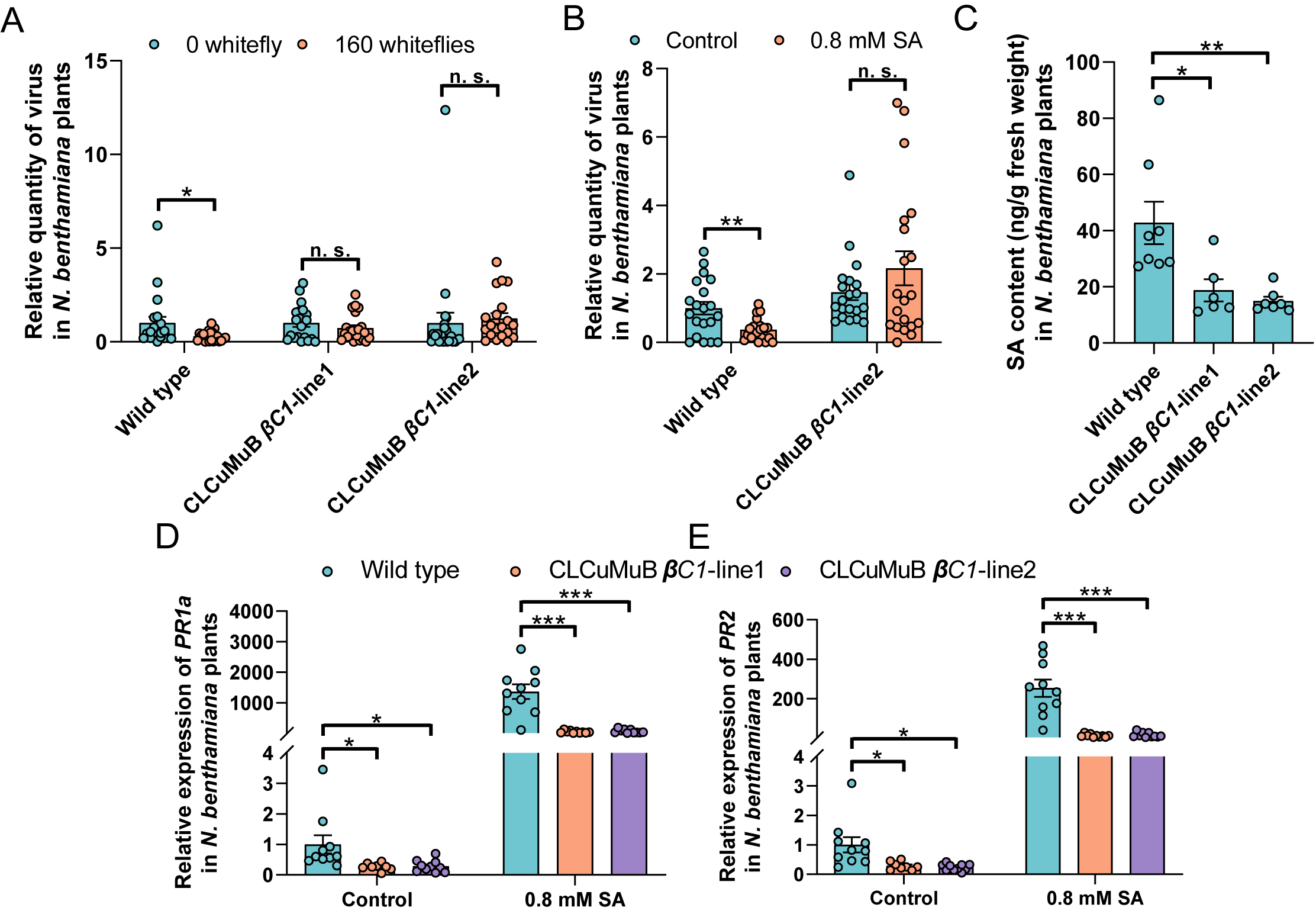

Supplement: S7 Fig — (A and B) Relative CLCuMuV quantity in wild type and CLCuMuB βC1-transgenic N. benthamiana plants that were first inoculated with CLCuMuV and then treated with whitefly (A) or SA (B); (C) SA content in wild type and CLCuMuB βC1-transgenic N. benthamiana plants; (D and E) Relative mRNA level of PR1a (D) and PR2 (E) in wild type and CLCuMuB βC1-transgenic plants. N = 20–22 plants for A-B, 6–8 samples (2–3 plants per sample) for C, 8–10 samples (2–3 plants per sample) for D-E. Data are mean ± SEM. n. s. stands for no significant difference, *P < 0.05, **P < 0.01, and ***P < 0.001 (two-sided Student’s t test). (TIF) [file ppat.1013907.s009.tif]

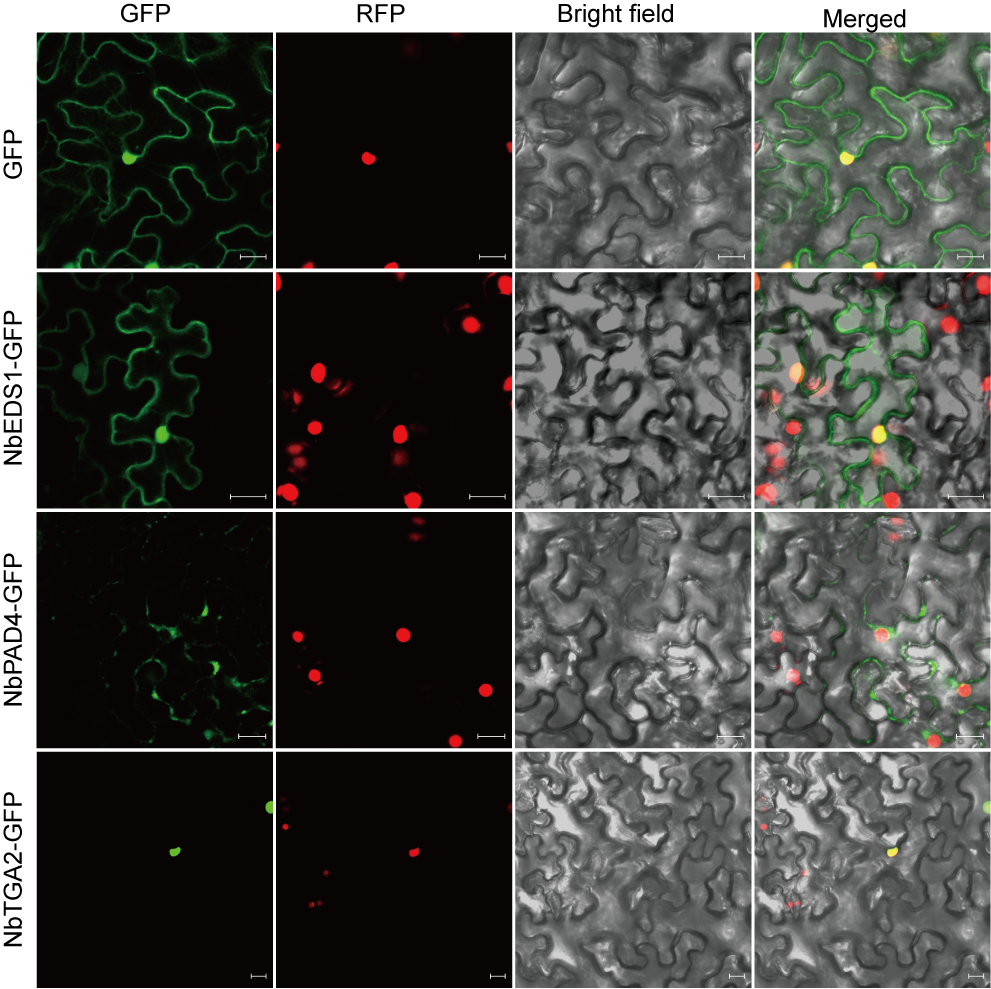

Supplement: S8 Fig — GFP, NbEDS1-GFP, NbPAD4-GFP and NbTGA2-GFP were expressed in the leaves of H2B-RFP transgenic N. benthamiana plants. Fluorescence was examined at two days post inoculation. (TIF) [file ppat.1013907.s010.tif]

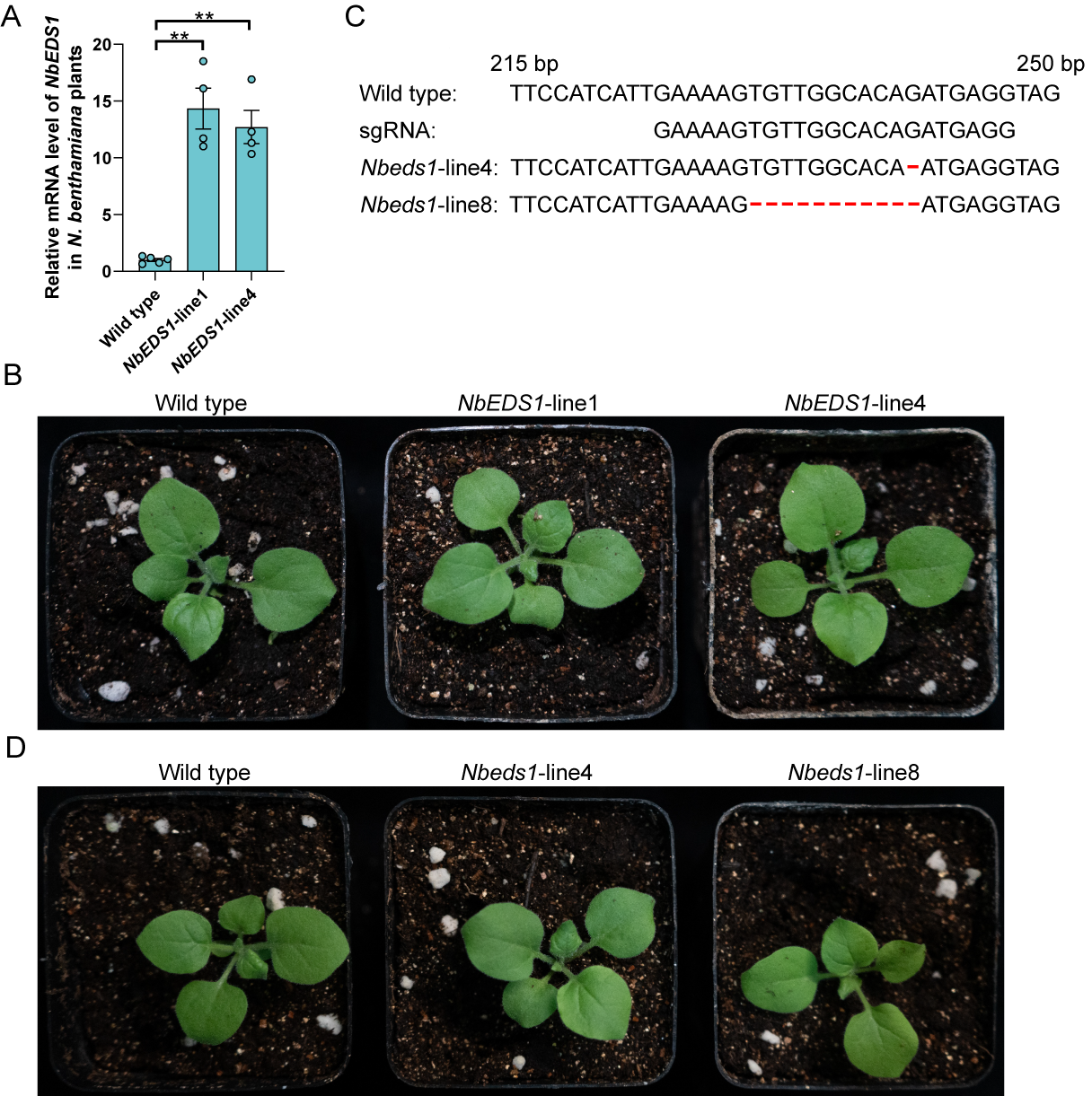

Supplement: S9 Fig — (A) Relative mRNA level of NbEDS1 in wild type and NbEDS1-transgenic N. benthamiana plants; (B) Sequences of NbEDS1 in wild type and Nbeds1 N. benthamiana plants. Red lines indicate deleted base pairs; (C and D) Wild type and NbEDS1-overexpressing (C) and knockout (D) plants. N = 4–5 samples (2–3 plants per sample) for A. Data are mean ± SEM. **P < 0.01 (two-sided Student’s t test). (TIF) [file ppat.1013907.s011.tif]

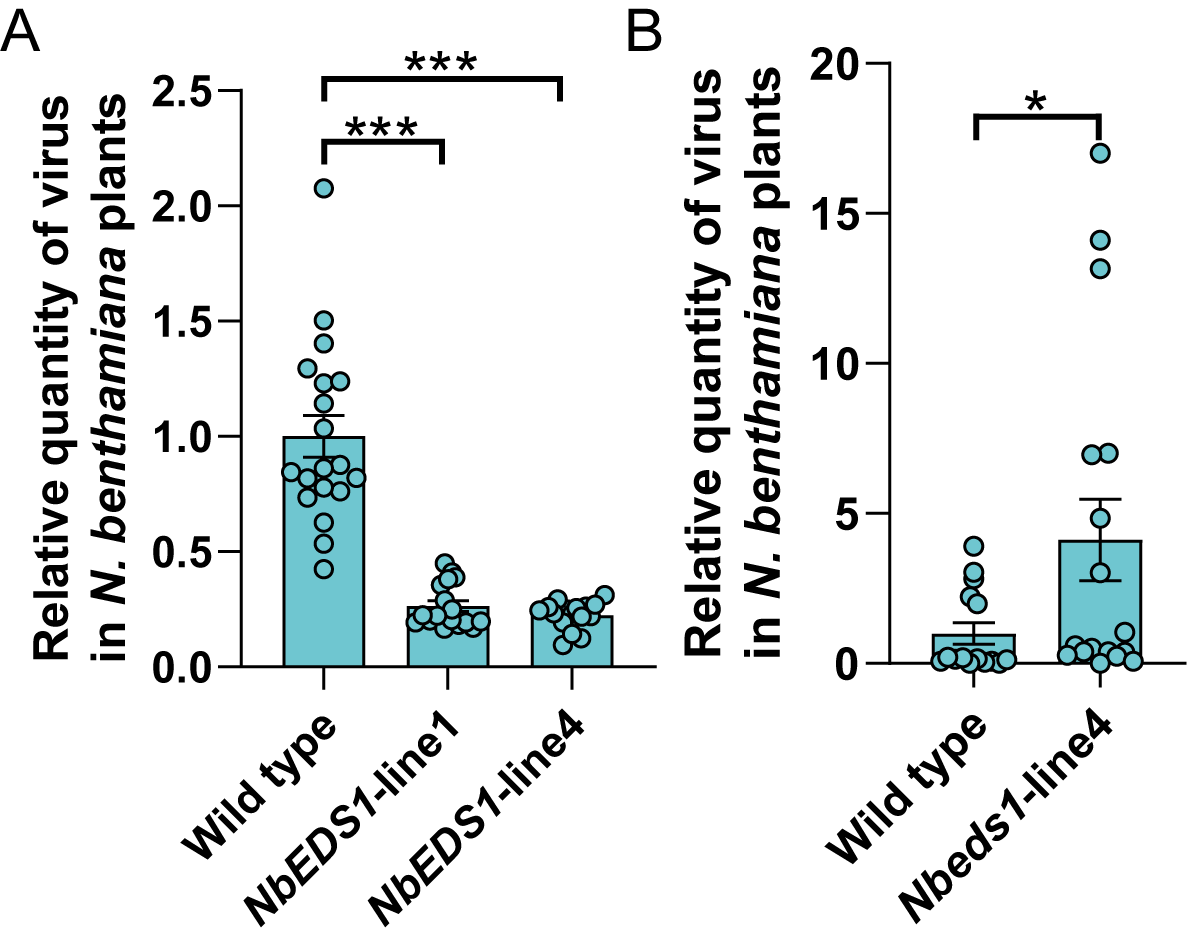

Supplement: S10 Fig — Wild type and NbEDS1-transgenic (NbEDS1) or NbEDS1-knockout (Nbeds1) N. benthamiana plants were inoculated with CLCuMuV + CLCuMuB. At ten days post inoculation, plants were sampled and subjected to the quantification of CLCuMuV. N = 15–19 plants. Data are mean ± SEM. *P < 0.05, and ***P < 0.001 (two-sided Student’s t test). (TIF) [file ppat.1013907.s012.tif]

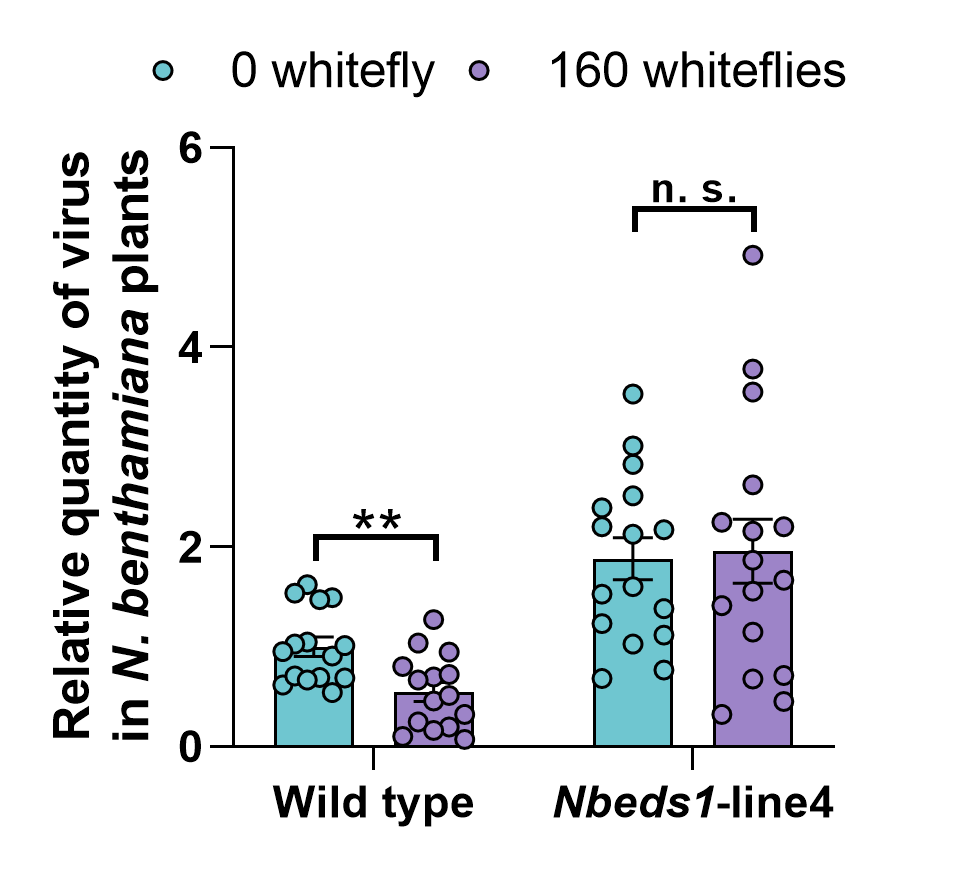

Supplement: S11 Fig — Wild type and NbEDS1-knockout (Nbeds1) N. benthamiana plants were inoculated with CLCuMuV and then subjected to whitefly infestation. N = 15–16 plants. Data are mean ± SEM. **P < 0.01 (two-sided Student’s t test). (TIF) [file ppat.1013907.s013.tif]

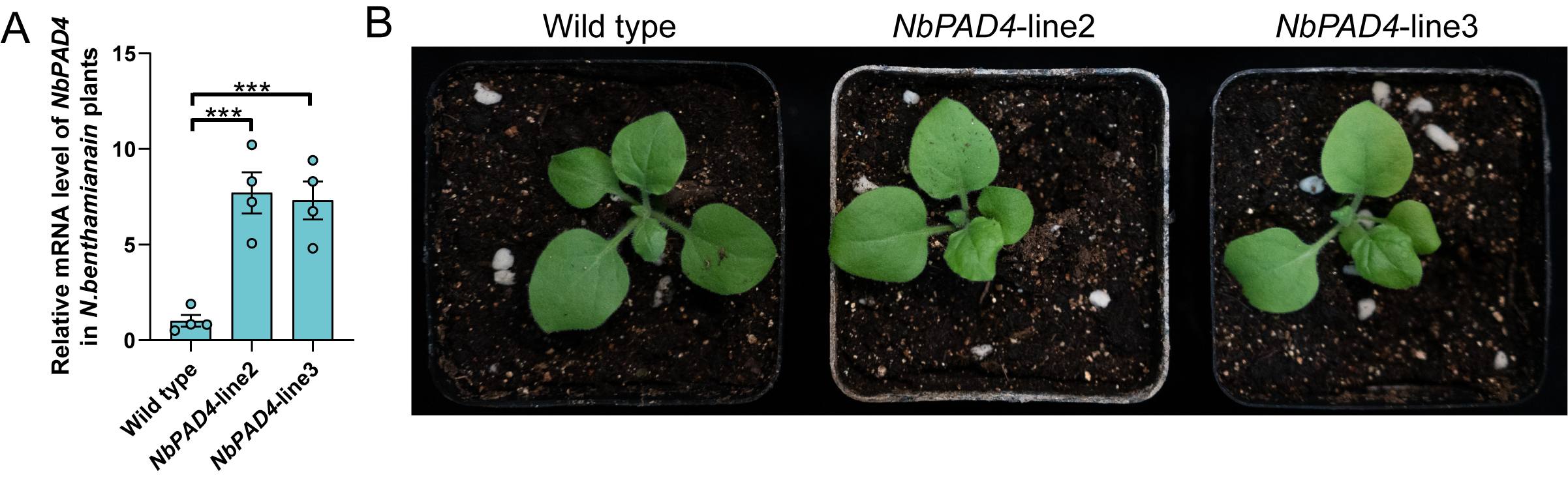

Supplement: S12 Fig — (A) Relative mRNA level of NbPAD4 in wild type and NbPAD4-transgenic N. benthamiana plants; (B) Picture of wild type and NbPAD4-overexpressing N. benthamiana plants. N = 4 samples (2–3 plants per sample) for a. Data are mean ± SEM. ***P < 0.001 (two-sided Student’s t test). (TIF) [file ppat.1013907.s014.tif]

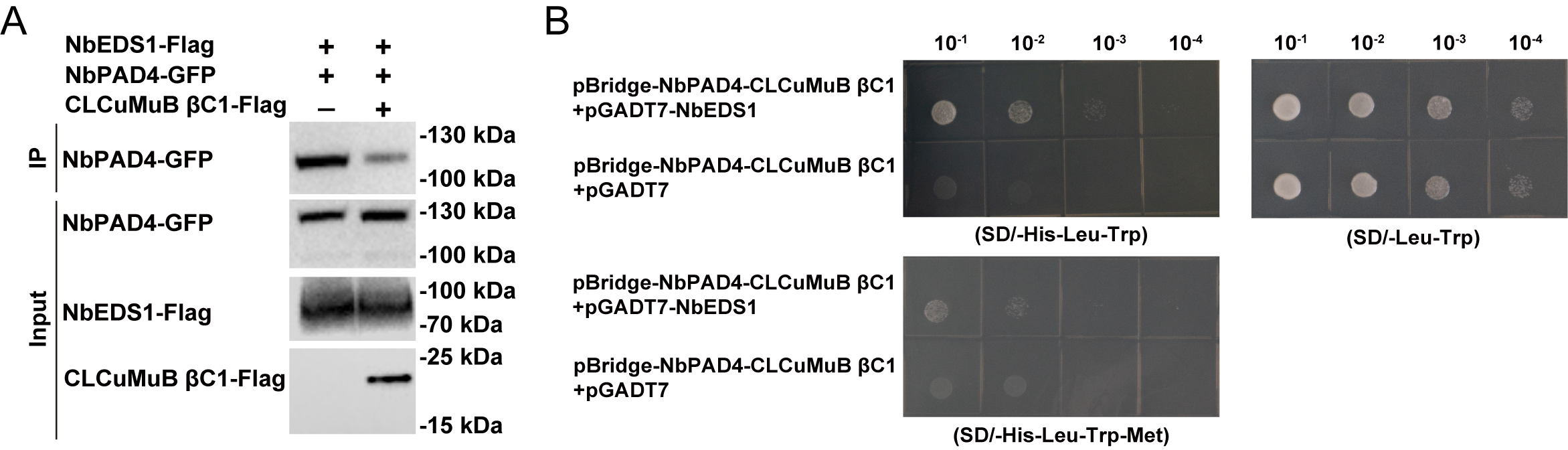

Supplement: S13 Fig — (A) Effect of CLCuMuB βC1 on NbEDS1-NbPAD4 interactions in co-IP assay. NbEDS1-Flag+NbPAD4-GFP was co-expressed with CLCuMuB βC1-Myc or Myc empty vector in the leaves of N. benthamiana plants. Proteins were extracted and subjected to immunoprecipitation with anti-Flag beads. (B) Effect of CLCuMuB βC1 on NbEDS1-NbPAD4 interactions in yeast three-hybrid assay. Yeast cells were transformed with pBridge-NbPAD4-CLCuMuB βC1 + pGADT7-NbEDS1 or pBridge-NbPAD4-CLCuMuB βC1 + pGADT7 (control) and then grown on SD/-Leu-Trp medium. Yeast cells were then cultured and transferred to SD/-His-Leu-Trp and SD/-His-Leu-Trp-Met media. (TIF) [file ppat.1013907.s015.tif]

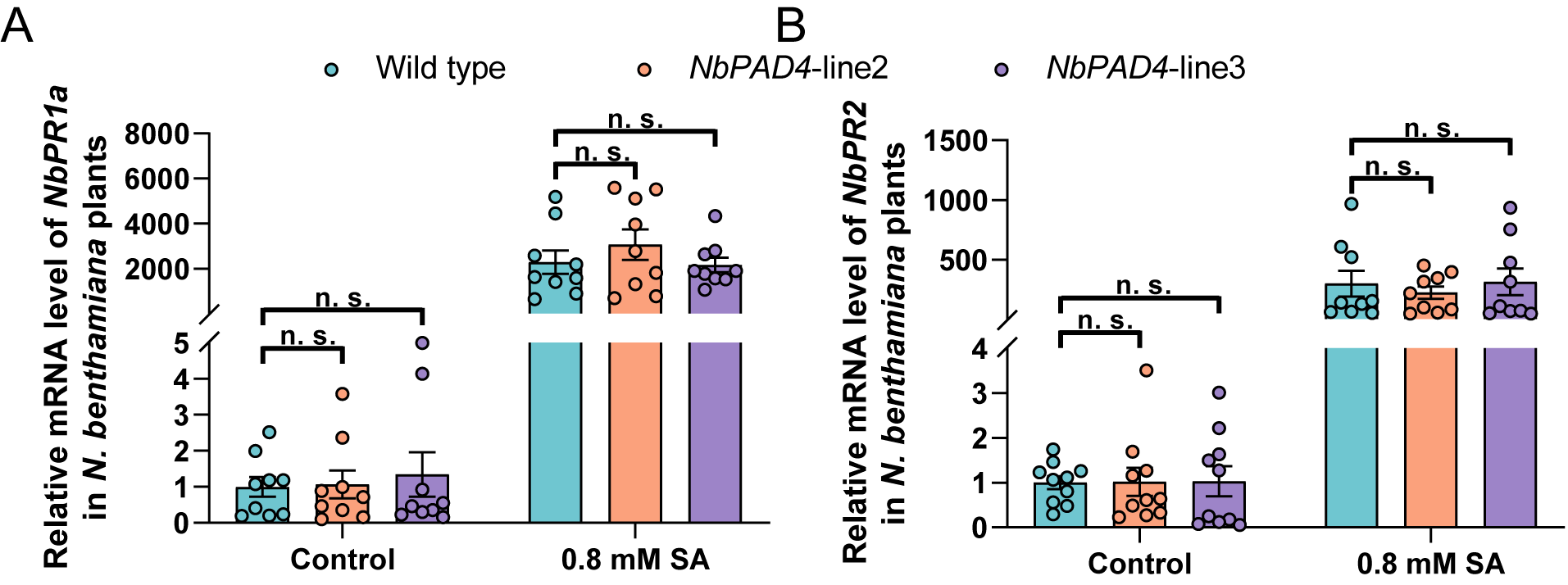

Supplement: S14 Fig — Wild type and NbPAD4-overexpressing N. benthamiana plants were treated with ethanol (control) or SA and then subjected to the transcriptional profiling of SA-responsive genes. N = 9–10 samples (2–3 plants per sample). Data are mean ± SEM. n. s. stands for no significant difference (two-sided Student’s t test). (TIF) [file ppat.1013907.s016.tif]

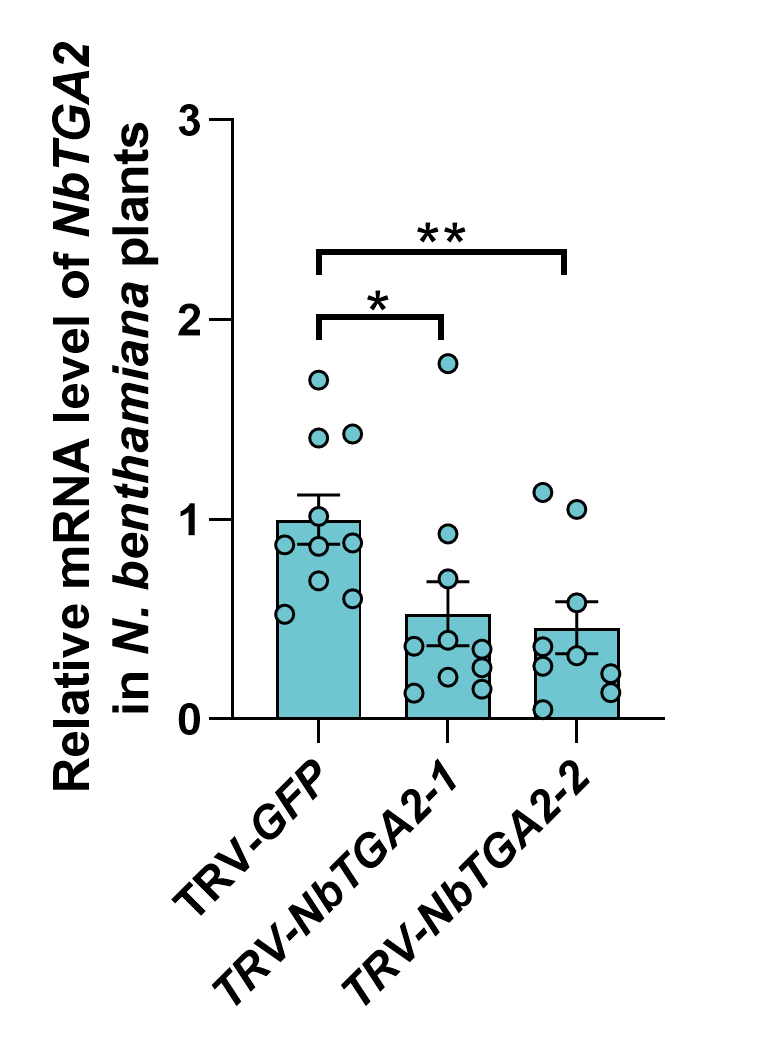

Supplement: S15 Fig — N. benthamiana plants were inoculated with TRV1 + TRV-GFP, TRV1 + TRV-NbTGA2–1, TRV1 + TRV-NbTGA2–2. At seven days post inoculation, plants were sampled and subjected to the analysis of NbTGA2 transcripts. N = 9–10 plants. Data are mean ± SEM. *P < 0.05, and **P < 0.01 (two-sided Student’s t test). (TIF) [file ppat.1013907.s017.tif]

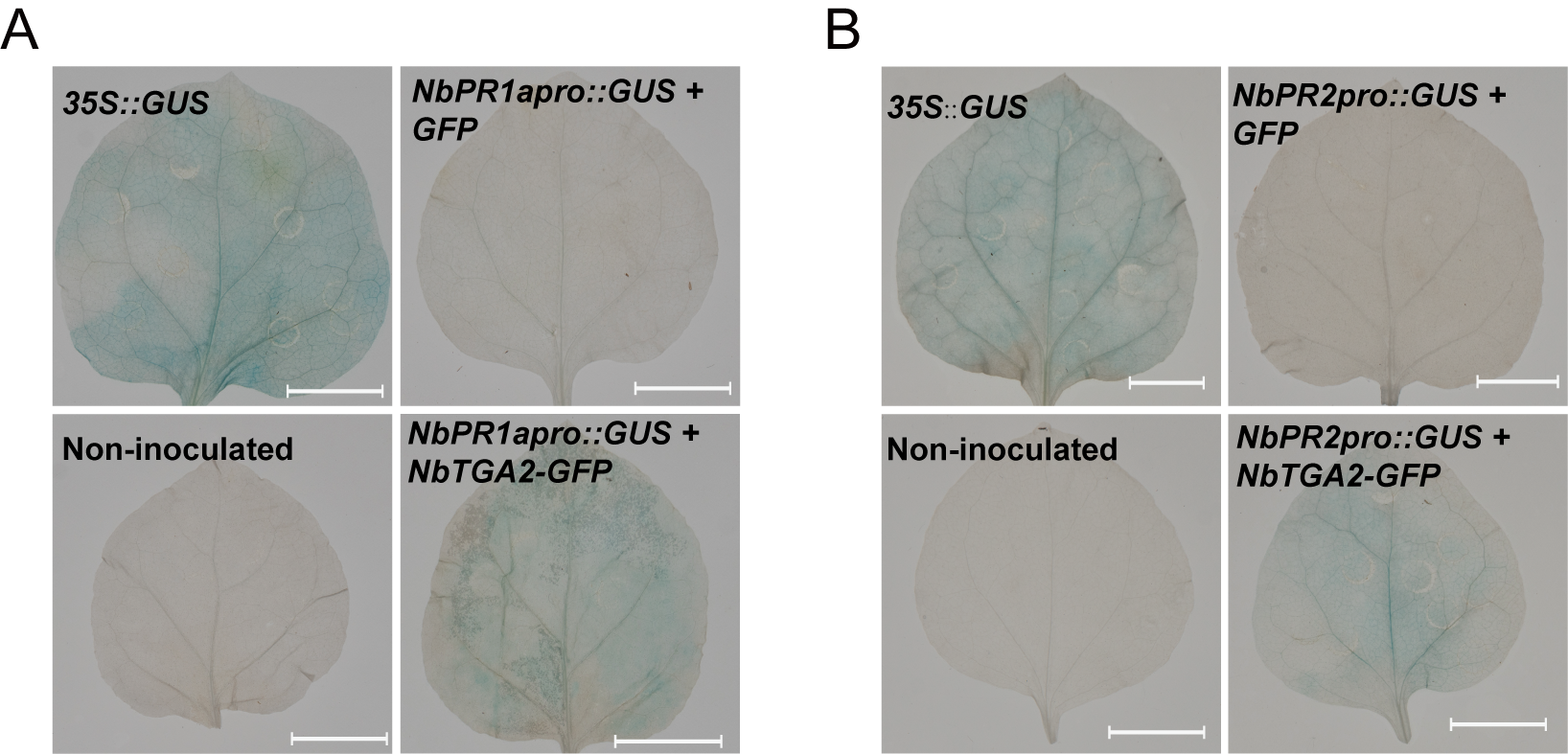

Supplement: S16 Fig — The promoter regions (2000 bp) of NbPR1a and NbPR2 were ligated into PBI121 to generate NbPR1apro-GUS and NbPR2pro-GUS. Recombinant plasmids were transformed into agrobacteria and then co-inoculated with GFP or NbTGA2-GFP into the leaves of N. benthamiana plants. Leaves inoculated with 35S-GUS were used as positive controls and non-inoculated leaves as negative controls. At two days post inoculation, leaves were harvested and subjected to GUS staining. (TIF) [file ppat.1013907.s018.tif]

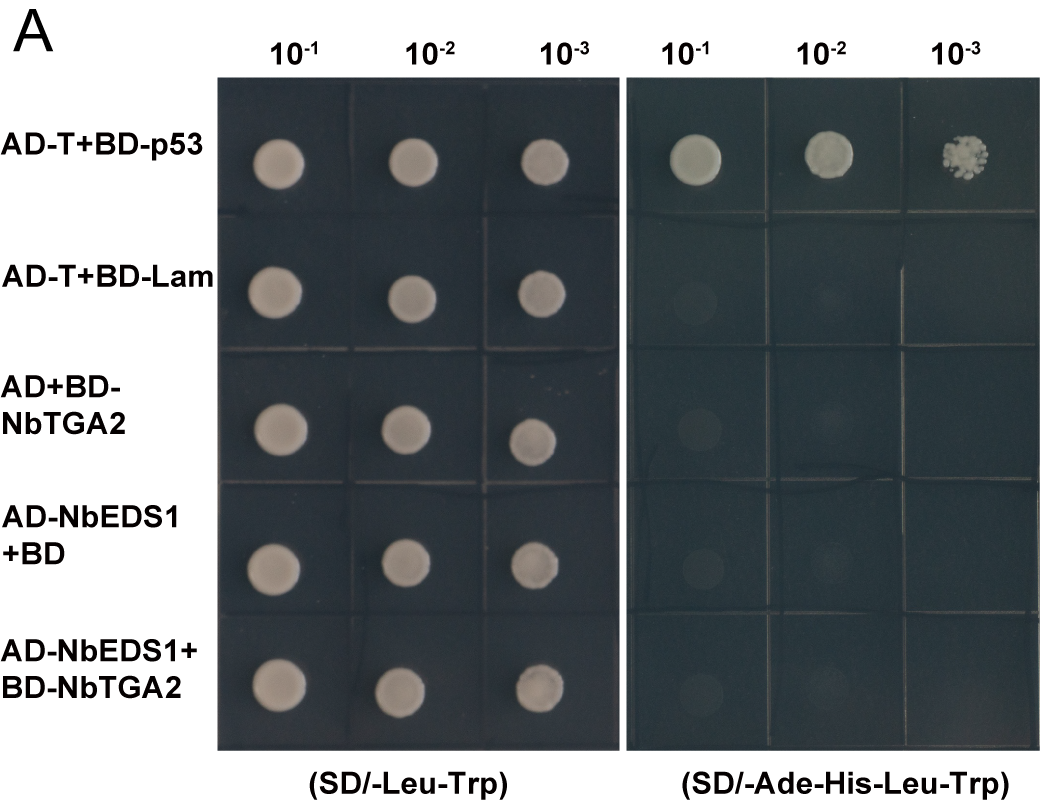

Supplement: S17 Fig — Yeast cells (strain AH109) were transformed with plasmid combinations (indicated on the left) and then spotted on selective media. (TIF) [file ppat.1013907.s019.tif]

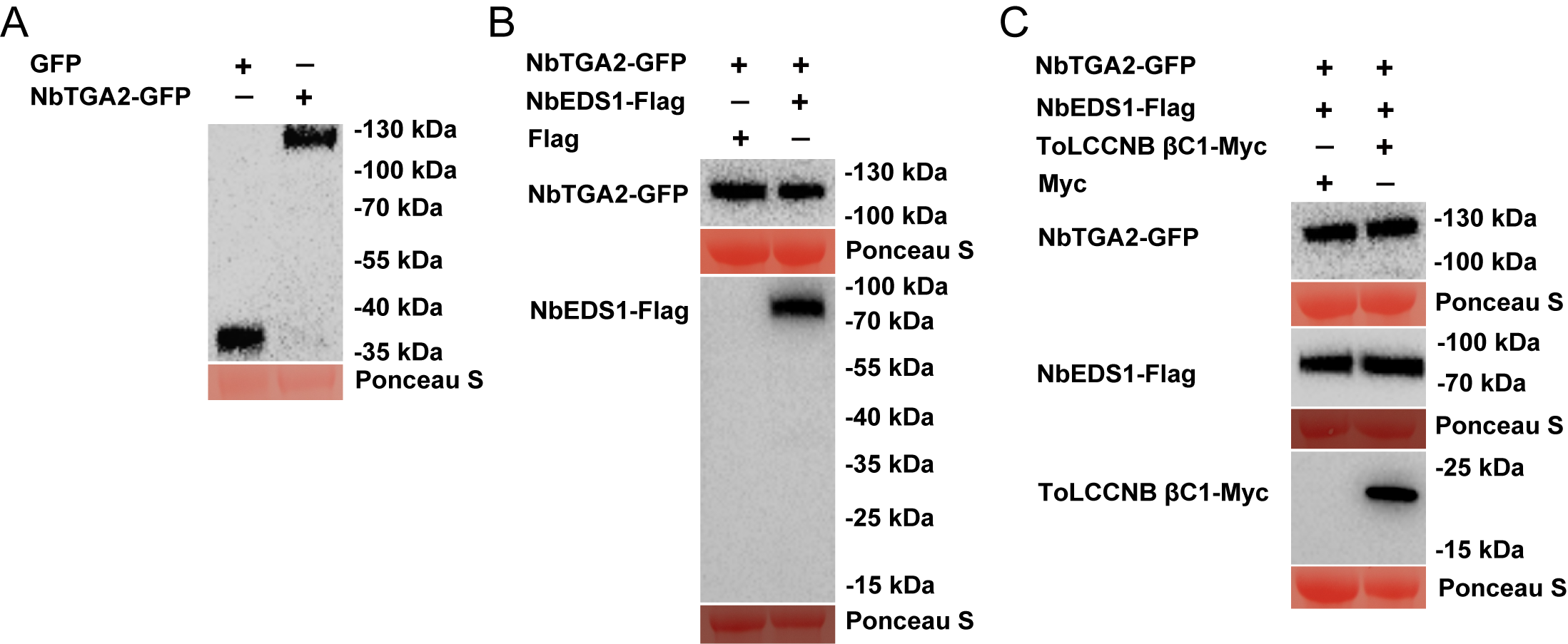

Supplement: S18 Fig — NbPR2pro-LUC was co-expressed with GFP, NbTGA2-GFP, Flag+NbTGA2-GFP, NbEDS1-Flag+NbTGA2-GFP, Myc + NbEDS1-Flag+NbTGA2-GFP, or ToLCCNB βC1 + NbEDS1-Flag+NbTGA2-GFP in the leaves of N. benthamiana plants. At two days post inoculation, leaves were harvested for western blotting. (TIF) [file ppat.1013907.s020.tif]
